# KHSRP-mediated decay of axonally localized prenyl-Cdc42 mRNA slows nerve regeneration

**DOI:** 10.1371/journal.pgen.1011916

**Published:** 2025-11-07

**Authors:** Matthew D. Zdradzinski, Lauren S. Vaughn, Samaneh Matoo, Kayleigh Trumbull, Terika P. Smith, Davis Noblitt, Courtney N. Buchanan, Ashley Loomis, Elizabeth Thames, Seung Joon Lee, Nora Perrone-Bizzozero, Qun Lu, Jessica M. Larsen, Jeffery L. Twiss

**Affiliations:** 1 Department of Biological Sciences, University of South Carolina, Columbia, South Carolina, United States of America; 2 Department of Chemical and Biomolecular Engineering, Clemson University, Clemson, South Carolina United States of America; 3 Genomic Medicine, Biogen, Cambridge, Massachusetts, United States of America; 4 Department of Neurosciences, University of New Mexico, Albuquerque, New Mexico, United States of America; 5 South Carolina SmartState Centers for Neurotherapeutics, University of South Carolina, Columbia, South Carolina, United States of America; 6 South Carolina SmartState Center for Childhood Neurotherapeutics, University of South Carolina, Columbia, South Carolina, United States of America; 7 Department of Pharmacology, Physiology and Neuroscience, University of South Carolina School of Medicine, Columbia, South Carolina, United States of America; 8 Carolina Autism and Neurodevelopment Center, University of South Carolina, Columbia, South Carolina United States of America; Harvard Medical School, UNITED STATES OF AMERICA

## Abstract

The small GTPase CDC42 promotes axon growth through actin filament polymerization and this growth is driven by axonal localization of the mRNA encoding the prenylated CDC42 isoform (*Prenyl-Cdc42*). Here, we show that axonal *Prenyl-Cdc42* mRNA levels and the mRNA’s translation are decreased by growth-inhibiting stimulation and increased by growth-promoting stimulation. In contrast, axonal *RhoA* mRNA transport and translation are increased by growth-inhibiting but unaffected by growth-promoting stimuli. Localized increase in KHSRP in response to growth inhibitory stimulation, through elevation of intracellular Ca^2+^, promotes decrease in axonal levels of *Prenyl-Cdc42* mRNA. Distinct 3’UTR motifs regulate transport and axonal levels of *Prenyl-Cdc42* mRNA. KHSRP protein binds to a *Prenyl-Cdc42* mRNA motif within nt 801–875 and the mRNA is remarkably increased in axons of *Khsrp*^*-/-*^ mice. Depletion of the mRNA from sciatic nerve indicates that the increased axonal Prenyl-CDC42 contributes to the accelerated nerve regeneration when neuronal KHSRP is depleted.

## Author summary

Regrowth of the axons making up peripheral nerves after traumatic injury is possible but the regeneration is too slow to return full functionality over anything beyond a few centimeters distance in the nerve. This results in loss of sensations and movements distal to the injury and can lead to pathological pain. Better understanding of the molecular processes that modulate regrowth after injury could lead to new therapeutic strategies for accelerating nerve regeneration and have benefits for brain and spinal cord injury where regeneration of axons completely fails. CDC42 proteins are known to promote axon growth, which was recently shown to be driven by localized synthesis of the prenylated CDC42 isoform in axons. We find that growth-promoting factors stimulate translation of the axonal CDC42 mRNA while growth-inhibiting stimuli decrease translation by promoting decay of the mRNA. The RNA binding protein KHSRP targets axonal CDC42 mRNA for degradation. KHSRP is known to slow axon regeneration and mice lacking KHSRP show accelerated nerve regeneration. We find that removing CDC42 mRNA from the injured axons of KHSRP knockout mice slows their axon regeneration, indicating that KHSRP-mediated decay of axonal CDC42 mRNA slows nerve growth.

## Introduction

Polymerization and depolymerization of actin filaments in axonal growth cones, through the activation of small Rho GTPases, promotes axon extension, turning, and retraction [1]. In growing axons, intracellular signaling cascades from attractant and repulsant stimuli differentially activate Rho GTPases, including RHOA, RAC1, and CDC42 locally in growth cones. Activation of RHOA promotes actin filament depolymerization while activation of RAC1 and CDC42 promotes actin filament polymerization [2]. Synthesis of proteins locally in axons has emerged as a mechanism that can impact axon growth, both during development and after injury [3]. Interestingly, the mRNAs encoding RHOA, RAC1, and a CDC42 isoform have been shown to localize into axons [4–6]. Translation of axonal *RhoA* mRNA in embryonic sensory neurons was initially reported in response to the growth inhibitory stimulus Semaphorin 3A, leading to growth cone collapse and axon retraction [6,7]. Conversely, nerve growth factor (NGF) activates axonal *Rac1* mRNA translation in developing sympathetic axons, with subsequent local prenylation of new RAC1 protein promoting axon extension [5]. The *Cdc42* RNA is differentially spliced to generate prenylated- or palmitoylated-CDC42 proteins (Prenyl-CDC42 and Palm-CDC42), respectively. We recently showed that the *Prenyl-Cdc42* mRNA, but not *Palm-Cdc42* mRNA, localizes into growing axons and is needed for axon growth [4]. Taken together, these findings point to primary roles for local synthesis of Rho GTPase proteins in axon extension and retraction but it is not clear if or how the stoichiometry of the encoded proteins is affected by the balance of growth-promoting/attractant *vs.* growth-inhibiting/repulsive stimuli.

*Palm-Cdc42* and *Prenyl-Cdc42* mRNAs are generated by differential inclusion of *CDC42* gene’s exons 6 and 7 [8]. Yap et al. (2016) reported that Palm-CDC42 (CDC42-Ex6) promotes dendritic spine development while Prenyl-CDC42 (CDC42-Ex7) promotes axon growth [9]. Exons 6 and 7 encode for different C-terminal 10 amino acids in their protein products and the transcripts have different 3’ untranslated regions (UTR). We recently showed that the different 3’UTRs are responsible for distinct subcellular localization of these *Cdc42* mRNA isoforms in cortical neurons, with *Palm-Cdc42* mRNA localizing selectively into dendrites and *Prenyl-Cdc42* mRNA localizing into axons and dendrites [4]. In dorsal root ganglion (DRG) sensory neurons that only extend axonal processes [10–12], *Prenyl-Cdc42* mRNA localizes into axons while *Palm-Cdc42* mRNA is retained in the cell body [4]. In both sensory and cortical neurons, axon growth promotion by CDC42 requires intra-axonal synthesis of Prenyl-CDC42. Here, we show that the *Prenyl-Cdc42* mRNA 3’UTR contains two distinct motifs that impact axonal CDC42 levels. The proximal 35 nucleotides (nt; 763–800) are needed for axonal localization, so this regulates how much of the mRNA localizes into axons. Just distal to that motif is a relatively adenine-uridine (AU)-rich sequence (nt 801–875). We previously showed that depletion of the axonal RNA binding protein (RBP) KH splicing regulatory protein (KHSRP) accelerates peripheral nerve regeneration [13]; we find that KHSRP binds to the AU-rich UTR region in *Prenyl-Cdc42* mRNA and decreases axonal levels of *Prenyl-Cdc42* mRNA. Axon growth-promoting stimulus, a cocktail of three neurotrophins that activates TrkA, B, and C receptors residing on most DRG neuronal subpopulations [14], increases levels and translation of axonal *Prenyl-Cdc42* mRNA in growing axons. The growth-inhibiting chondroitin sulfate proteoglycan (CSPG) aggrecan decreases *Prenyl-Cdc42* mRNA levels and translation in axons but simultaneous increases axonal RHOA and KHSRP proteins. Together, our findings suggest that axonal KHSRP slows axon growth by decreasing of axonal *Prenyl-Cdc42* mRNA levels. This is supported by depletion of *Prenyl-Cdc42* mRNA slowing axon regeneration in *KHSRP* knockout mice.

## Results

### Extracellular stimuli can regulate axonal levels and translation of Prenyl-Cdc42 and RhoA mRNAs

**Fig 1:**
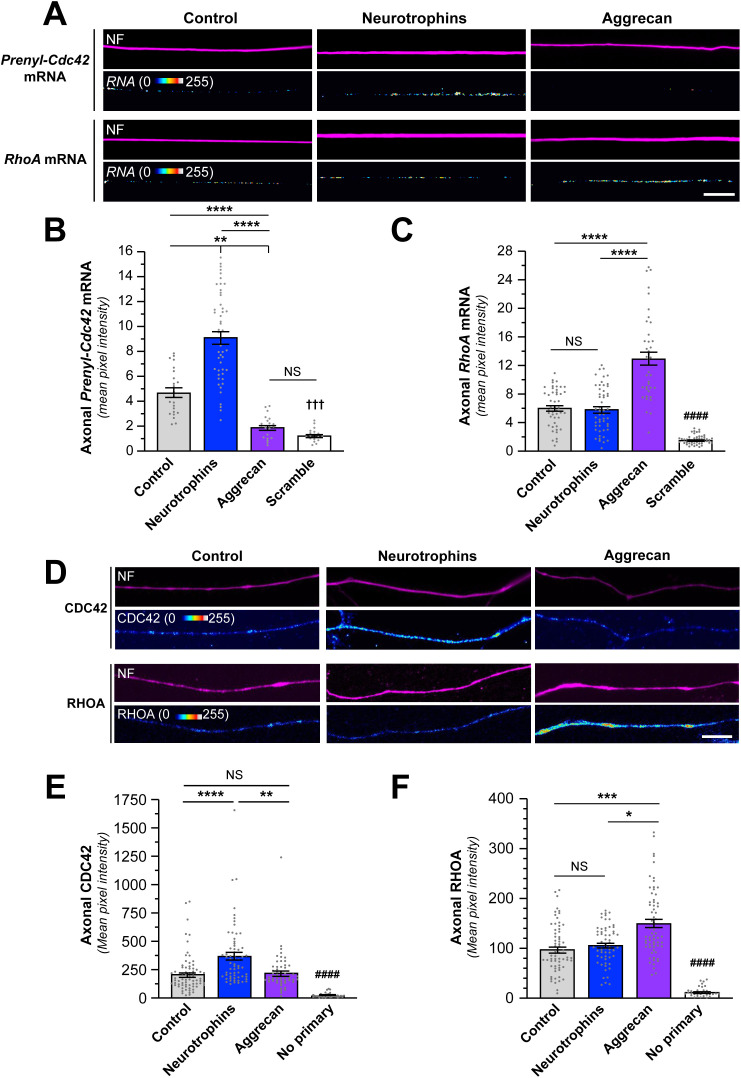
Growth-promoting and inhibiting stimuli regulate axonal levels of Prenyl-Cdc42 and RhoA mRNAs. **A)** Representative exposure-matched smFISH and IF images for *Prenyl-Cdc42* or *RhoA* mRNA plus neurofilament (NF) protein in adult DRG neuron cultures treated with either 10 ng/ml each NT3, BDNF, plus NGF each (‘neurotrophins’) or 50 ng/ml aggrecan [Scale bar = 10 µm]. **B-C)** Quantification of axonal *Cdc42* (B) or *RhoA* (C) mRNA smFISH signal intensities shown as mean pixel intensity above background for axons ± SEM (*N* ≥ 40 neurons across three independent cultures; ** P < 0.01, **** P < 0.001 and NS = not significant for indicated data sets, †††† P < 0.001 vs. control and neurotrophins, and #### P < 0.001 vs. aggrecan by Kruskal-Wallis ANOVA with Dunn post-hoc tests for pair-wise comparisons). **D)** Representative exposure-matched IF images for Prenyl-CDC42 or RHOA proteins and NF in distal axons of adult DRG neuron cultures treated with either 10 ng/ml neurotrophins or 50 ng/ml aggrecan; see S1A Fig for representative no primary IF images [Scale bar = 10 µm]. **E-F)** Quantitation of axonal CDC42 (E) or RHOA **(F)** IF signal intensities shown as mean pixel intensity above background ± SEM; see S1B and S1C Fig for cell body levels of CDC42 and RHOA under these conditions (*N* ≥ 20 neurons in three independent cultures; * P < 0.05, ** P < 0.01, *** P < 0.005, **** P < 0.001, and NS = not significant between indicated pairs and #### P < 0.001 vs. all other groups by Kruskal-Wallis ANOVA with Dunn post-hoc tests for pair-wise comparisons).

We previously found that localized translation of *Prenyl-Cdc42* mRNA promotes neurite growth in cultured neurons [4]. Axon growth can be positively or negatively impacted by external stimuli, so we asked if growth-promoting neurotrophins or a growth-inhibiting CSPG might alter axonal transport of *Prenyl-Cdc42* mRNA by treating mouse DRG cultures with a neurotrophin cocktail, consisting of NT3, BDNF, and NGF to stimulate all 3 Trk receptors on DRG neuron subpopulations, or aggrecan and assessed axonal *Prenyl-Cdc42* mRNA levels by single molecule fluorescent *in situ* hybridization (smFISH). Since DRG neurons can be cultured from adult mice, this model provides a view of axons regrowing from a mature neuron where we previously showed *Prenyl-Cdc42* mRNA localizes. Neurotrophin treatment increased and aggrecan decreased axonal *Prenyl-Cdc42* mRNA levels (Fig 1A and 1B). In contrast to CDC42’s promotion of actin filament polymerization, RHOA activation causes actin filament depolymerization and the protein’s activity is increased by growth-inhibiting stimuli [2]. *RhoA* mRNA also localizes into axons, and this was shown to increase upon exposure to CSPGs [15]; consistent with this, we see that axonal *RhoA* mRNA levels increase in response to aggrecan but neurotrophin stimulation had no effect on axonal *RhoA* mRNA levels (Fig 1A and 1C). Together, these data point to reciprocal regulation of axonal *RhoA* and *Prenyl-Cdc42* mRNAs in response to CPSG stimulation but selective increase in axonal *Prenyl-Cdc42* mRNA in response to neurotrophins.

To determine if the endogenous Prenyl-CDC42 and RHOA proteins might show similar changes in their axonal levels with these stimuli, we performed immunofluorescence analyses on DRG cultures that had been treated with neurotrophins or aggrecan. Exposure to neurotrophins increased axonal CDC42 protein levels but had no apparent effect on axonal RHOA levels (Fig 1D and 1F). Aggrecan treatment increased RHOA protein levels in the axons but had no apparent effect on axonal CDC42 protein levels (Fig 1D-E). CDC42 protein levels did not change in the neuronal cell bodies or soma with these stimuli (S1A Fig). Soma RHOA protein levels increased with both aggrecan and neurotrophin treatments (S1B Fig). It should be noted that the CDC42 antibody utilized here does not distinguish between Prenyl-CDC42 and Palm-CDC42 proteins. We previously showed that Palm-CDC42 protein is transported into axons [4], so these immunofluorescence data do not distinguish between the two CDC42 isoforms.

**Fig 2:**
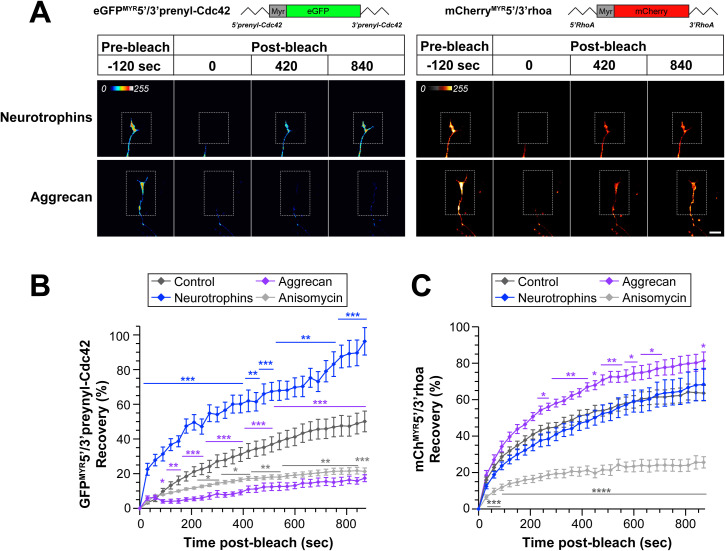
Growth-promoting and inhibiting stimuli regulate axonal translation of Prenyl-Cdc42 and RhoA mRNAs. **A)** Representative FRAP image sequences for DRG neurons co-transfected with GFP^MYR^5’/3’prenyl-Cdc42 and mCherry^MYR^5’/3’RhoA are shown (72 h post-transfection). Cultures were treated with either 10 ng/ml neurotrophins or 50 ng/ml aggrecan as in Fig 1. Boxed regions represent the photobleached ROIs; see S2A and S2B Fig for control and anisomycin-treated representative FRAP image sequences [Scale bar = 20 µm]. **B-C)** Quantitation of FRAP sequences from panel A are shown as average normalized% recovery ± SEM. Note that translation inhibition with anisomycin prior to photobleaching shows that the GFP^MYR^5’/3’prenyl-Cdc42 and mCherry^MYR^5’/3’RhoA recovery requires protein synthesis (*N* ≥ 10 neurons over three independent experiments; * *P* < 0.05, ** P < 0.01, ***P < 0.005, ****P < 0.001 by two-way repeated measures ANOVA with Tukey post-hoc tests for pair-wise comparisons).

To more selectively assess axonal translation of *Prenyl-Cdc42* and *RhoA* mRNAs in response to these stimuli, we visualized axonal signals of diffusion limited GFP^MYR^ and mCherry^MYR^ reporters with the 5’ and 3’UTRs of rat *Prenyl-Cdc42* and *RhoA* mRNAs, respectively, as surrogates for local translation of the endogenous axonal mRNAs (GFP^MYR^5’/3’prenyl-Cdc42, mCherry^MYR^5’/3’RhoA). The 5’ and 3’UTRs were included to ensure that we captured both axonal localizing and any translational control motifs; the MYR tag limits diffusion of the newly synthesized GFP and mCherry proteins in neurites such that fluorescence recovery after photobleaching (FRAP) can be used to visualize sites of reporter mRNA translation [4,16,17]. FRAP analyses in DRG neurons co-expressing *GFP*^*MYR*^*5’/3’prenyl-Cdc42* and *mCherry*^*MYR*^*5’/3’RhoA* mRNAs showed fluorescent recovery within 15 min post-bleach that was attenuated by pretreatment with the protein synthesis inhibitor anisomycin, consistent with intra-axonal translation of the reporter mRNAs (Figs 2B, 2C, S2A and S2B). To determine if neurotrophin or aggrecan stimulation might affect translation of the *GFP*^*MYR*^*5’/3’prenyl-Cdc42* and *mCherry*^*MYR*^*5’/3’RhoA* mRNAs, we bath applied the neurotrophin cocktail or aggrecan for 30 min before photobleaching. Neurotrophin stimulation increased *GFP*^*MYR*^*5’/3’prenyl-Cdc42* mRNA translation in distal axons but, consistent with the axonal mRNA analyses above, had no effect on axonal *mCherry*^*MYR*^*5’/3’RhoA* mRNA translation (Fig 2A-C). In contrast, aggrecan treatment decreased *GFP*^*MYR*^*5’/3’prenyl-Cdc42* and increased *mCherry*^*MYR*^*5’/3’RhoA* translation in terminal axons (Fig 2A-C). The recovery of axonal GFP^MYR^5’/3’prenyl-Cdc42 fluorescence in the presence of aggrecan was largely indistinguishable from treatments with the protein synthesis inhibitor anisomycin (Figs 2A, 2B, S2A and S2B). Taken together, the data in Figs 1 and 2 are consistent with reciprocal regulation of axonal *Prenyl-Cdc42* and *RhoA* mRNA levels and subsequent intra-axonal translation in response to aggrecan. In contrast, responsiveness to neurotrophins appears to be limited to the axonal *Prenyl-Cdc42* mRNA.

**Fig 3:**
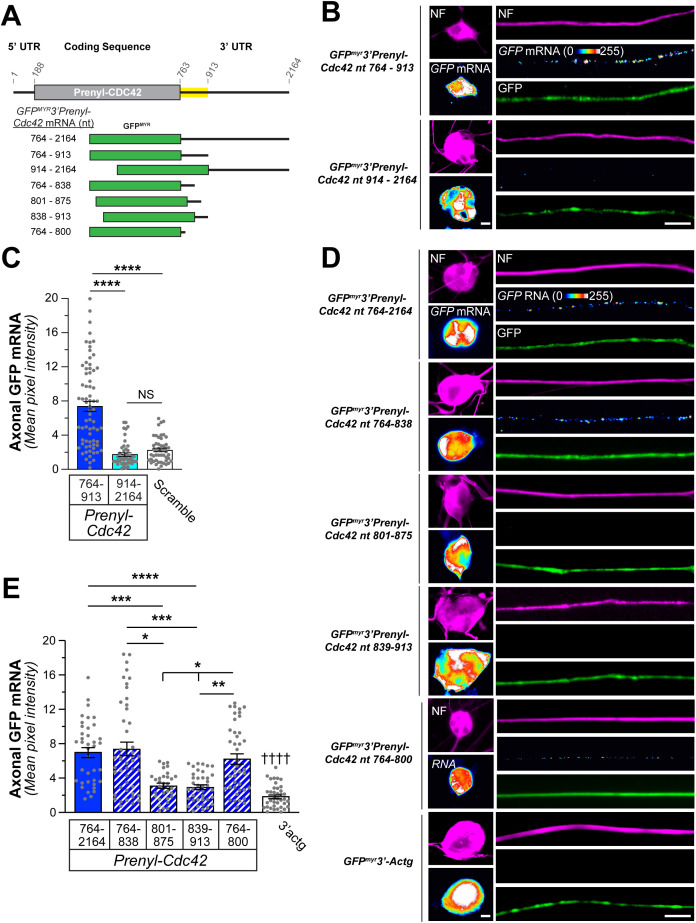
An evolutionarily conserved region of the Prenyl-Cdc42 mRNA 3’ UTR drives its axonal localization. **A)** Schematic of the regions of the rat *prenyl*-*Cdc42* 3′UTR mRNA that were tested for axonal localizing activity. The yellow-shaded regions show ≥85% sequence identity between available mammalian *prenyl*-*Cdc42* mRNAs (see S3 Fig for sequence alignments across species). GFP^MYR^ constructs used for testing 3’UTR segment are shown with GFP in green and RNA segment in black. **B)** Representative exposure-matched smFISH and immunofluorescence (IF) images for GFP^myr^ mRNA and neurofilament (NF) in adult DRG neuron cultures transfected with GFP^MYR^3’prenyl-Cdc42^764-913^, and GFP^MYR^3’prenyl-Cdc42^914-2164^. See S4A Fig for representative images of scrambled smFISH probe [Scale bar = 10 µm]. **C)** Quantitation of smFISH signal intensities shown as mean ± SEM pixel intensity above background for axons; see S4B Fig for cell body levels under these conditions (*N* ≥ 45 neurons across three independent cultures; **** P < 0.001 and NS = not significant as indicated comparisons by Kruskal-Wallis ANOVA with Dunn post-hoc tests for pair-wise comparisons). **D)** Representative exposure-matched smFISH and IF images for GFP mRNA plus NF in adult DRG neuron cultures transfected with GFP^MYR^3’prenyl-Cdc42^764-2164^, GFP^MYR^3’prenyl-Cdc42^764-838^, GFP^MYR^3’prenyl-Cdc42^801-875^, GFP^MYR^3’prenyl-Cdc42^839-913^, GFP^MYR^3’prenyl-Cdc42^764-800^ or GFP^MYR^3’actg [Scale bar = 10 µm]. **E)** Quantitation of smFISH signal intensities shown as mean ± SEM pixel intensity above background for axons; see S4C Fig for cell body levels under these conditions (*N* ≥ 40 neurons across three independent cultures; * *P* < 0.05, ** *P* < 0.01, *** *P* < 0.005, **** *P* < 0.001 for indicated data sets and †††† *P* < 0.001 vs. all but 801-875 and 839-913 by Kruskal-Wallis ANOVA with Dunn post-hoc tests for pair-wise comparisons).

As noted above, *Prenyl-Cdc42* mRNA localizes into axons of sensory neurons and cortical neurons and the 3’UTR encoded by *CDC42* exon 7 is necessary and sufficient for the mRNA’s axonal localization [4]. We have previously shown that conservation of 3’UTR regions can have predictive value for identifying functional domains in mRNAs [18,19]. The initial 150 nt of rat *Prenyl-Cdc42* 3’UTR (nt 764–913; NCBI XM_008764286.3) shows > 85% sequence identity with the 3’UTRs of 26 other vertebrate species (S3 Fig). To determine if this conserved 150 nt region imparts any function for the mRNA, we generated fluorescent reporter constructs containing *Prenyl-Cdc42*’s nt 764–913 or 914–2164 (*i.e.*, the remainder of the 3’UTR) as the 3’UTR for GFP^MYR^ cDNA (GFP^MYR^3’prenyl-Cdc42^764-913^ and GFP^MYR^3’prenyl-Cdc42^914-2164^, respectively; Fig 3A). smFISH analyses of transfected adult mouse DRG cultures showed that *GFP*^*MYR*^ mRNA only localized into axons of the GFP^MYR^3’prenyl-Cdc42^764-913^ transfected neurons; GFP^MYR^3’prenyl-Cdc42^914-2164^ transfected neurons did not show axonal *GFP* mRNA signal above the scrambled control probe (Figs 3B, 3C, and S4A). Cell body levels of *GFP* mRNA were not appreciably different between GFP^MYR^3’prenyl-Cdc42^764-913^ and GFP^MYR^3’prenyl-Cdc42^914-2164^ transfected neurons (Figs 3B and S4B). These data show that the conserved proximal 150 nt region of *Prenyl-Cdc42*’s 3’UTR is necessary and sufficient to drive axonal mRNA localization.

**Fig 4:**
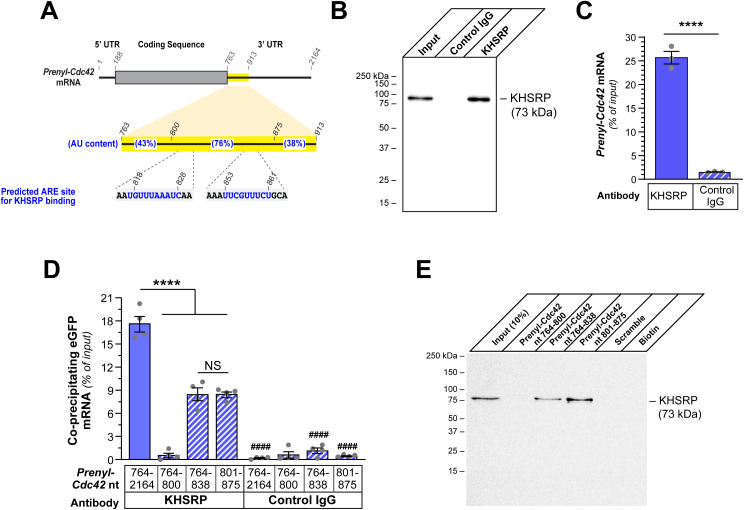
ARE-binding protein KHSRP binds to non-localizing conserved region of Prenyl-Cdc42 3’UTR. **A)** Schematic showing % adenine-uridine percentages across the conserved *Prenyl-Cdc42* mRNA nt 764-913 indicated in Fig 3A with predicted AREs for possible KHSRP binding indicated. **B-C)** Representative western blot for KHSRP protein (**B**) using KHSRP *vs*. control IgG immunoprecipitation from adult DRG cultures. RTddPCR analyses of RNA isolated from KHSRP vs. control IgG immunoprecipitates; co-precipitating *Prenyl-Cdc42* mRNA is shown as mean mRNA copies as percentage of input ± SEM (**C**; *N* = 3 biological replicates; **** *P* < 0.001 by Student’s *t*-*t*est for the indicated data pairs). **D)** Analyses of *GFP* mRNA from KHSRP and control IgG immunoprecipitates from DRG neuron cultures transfected with GFP^MYR^3’prenyl-Cdc42^764-2164^, GFP^MYR^3’prenyl-Cdc42^764-800^, GFP^MYR^3’prenyl-Cdc42^764-838^, or GFP^MYR^3’prenyl-Cdc42^801-875^ is shown as mean of co-precipitating mRNA copies as percentage of input ± SEM (*N* = 3 biological replicates; **** *P* ≤ 0.001 for indicated data pairs and #### *P* ≤ 0.001 for control IgG compared to corresponding KHSRP RIP by ordinary one-way ANOVA with Tukey post-hoc tests for pair-wise comparisons). **E)** Representative immunoblot analysis for KHSRP protein in RNA affinity pull down using biotinylated oligonucleotides corresponding to nt 764-838, 801-875 and 764-800 of rat *Prenyl-Cdc42* mRNA. Scrambled oligonucleotide and biotin alone were used as negative controls.

*Prenyl-Cdc42* mRNA’s nt 801–875 contains more than 75% adenine and uridine bases (see Fig 4A), which could contain an AU-rich element (ARE). The ARE in *Gap43* mRNA’s 3’UTR is necessary and sufficient for its localization into rat sensory axons and requires the ARE-binding protein HuD [20]. Thus, we asked which sequences within *Prenyl-Cdc42* nt 764–913 are sufficient for axonal localization of the mRNA. For this, we generated GFP^MYR^ reporter constructs containing 3 overlapping 75 nt portions of the 764–913 sequence (GFP^MYR^3’prenyl-Cdc42^764-838^, GFP^MYR^3’prenyl-Cdc42^801-875^, and GFP^MYR^3’prenyl-Cdc42^839-913^; Fig 3A). DRGs expressing the *GFP*^*MYR*^*3’prenyl-Cdc42*^*764-838*^ mRNA showed axonal localization of *GFP* mRNA by smFISH, but axonal *GFP* mRNA signals in the *GFP*^*MYR*^*3’prenyl-Cdc42*^*801-875*^ and *GFP*^*MYR*^*3’prenyl-Cdc42*^*839-913*^ expressing neurons were not distinguishable from neurons transfected with GFP^MYR^ containing a non-localizing 3’UTR (GFP^MYR^3’Actg; Fig 3D and 3E). Cell body levels of *GFP* mRNA were not significantly different between GFP^MYR^3’prenyl-Cdc42^764-838^, GFP^MYR^3’prenyl-Cdc42^801-875^, GFP^MYR^3’prenyl-Cdc42^839-913^, and GFP^MYR^3’actg transfected neurons (Figs 3D and S4C).

Since nt 764–838 of *Prenyl-Cdc42* mRNA contains an AU rich area over nt 801–838 (~76% AU compared to 43% for nt 764–800), we asked if the nt 764–800 has any localizing activity on its own. Thus, we generated a fluorescent reporter construct containing *Prenyl-Cdc42*’s 3’UTR nt 764–800 (GFP^MYR^3’prenyl-Cdc42^764-800^; Fig 3A). DRG neurons expressing *GFP*^*MYR*^*3’Prenyl-Cdc42*^*764-800*^ mRNA showed robust axonal *GFP* mRNA FISH comparable to *GFP*^*MYR*^*3’prenyl-Cdc42*^*764-838*^ mRNA expressing neurons (Figs 3E and S4C). Thus, the axonal localization motif in *Prenyl-Cdc42* mRNA lies in the most proximal 37 nt of its 3’UTR (nt 764–800).

### ARE-binding protein KHSRP binds to non-localizing conserved region of Prenyl-Cdc42 3’UTR

The decrease in axonal *Prenyl-Cdc42* mRNA in response to aggrecan coupled with the presence of an AU rich nature of the 3’UTR raise the possibility that ARE-binding proteins might impact *Prenyl-Cdc42* mRNA levels in axons (Fig 4A). KHSRP binds to ARE-containing mRNAs and promotes their decay by targeting those transcripts to the cytoplasmic exosome [21]. Thus, we tested whether KHSRP might bind to the endogenous *Prenyl-Cdc42* mRNA using RNA co-immunoprecipitation (RIP). The Anti-KHSRP antibody was first validated for immunoprecipitation of KHSRP protein by immunoblotting (Fig 4B). RNA isolated from these KHSRP immunoprecipitates showed that approximately 25% of input *Prenyl-Cdc42* mRNA co-precipitated with KHSRP by reverse transcriptase-coupled droplet digital PCR (RTddPCR; Fig 4C). Thus, endogenous KHSRP can bind to *Prenyl-Cdc42* mRNA in PNS DRGs.

The 3’UTR of *GFP*^*MYR*^*3’prenyl-Cdc42*^*801-875*^ mRNA is quite AU-rich and contains predicted ARE binding sites for KHSRP and HuD at nt 818–828 and 853–861, based on previously established consensus sequences [22,23] (Fig 4A). Thus, we asked if *Prenyl-Cdc42* mRNA nt 801–875 is bound by KHSRP. For this, we performed RIP analyses from DRG cultures transfected with the GFP^MYR^3’prenyl-Cdc42^764-2164^, GFP^MYR^3’prenyl-Cdc42^764-800^, GFP^MYR^3’prenyl-Cdc42^764-838^, or GFP^MYR^3’prenyl-Cdc42^801-875^ expression constructs. RTddPCR analyses of the immunoprecipitates showed that *GFP*^*MYR*^ mRNAs containing *Prenyl-Cdc42* mRNA nt 764–2164, 764–838, and 801–875, but not 764–800, were precipitated by anti-KHSRP antibodies (Fig 4D). IgG control showed no significant precipitation of *GFP*^*MYR*^ mRNA for any of the DRG transfectants (Fig 4D). The levels of *GFP*^*MYR*^ mRNA coprecipitating with KHSRP in the *GFP*^*MYR*^*3’prenyl-Cdc42*^*764-838*^ and *GFP*^*MYR*^*3’prenyl-Cdc42*^*801-875*^ mRNA expressing DRG cultures were about half of what was seen in the *GFP*^*MYR*^*3’prenyl-Cdc42*^*764-2164*^ mRNA expressing cultures suggesting that KHSRP’s interaction with this 3’UTR region may be affected by sequences downstream of nt 875 (Fig 4D). Consistent with this, ARE-like sequences are present downstream of nt 864 in the *Prenyl-Cdc42* mRNA 3’UTR (*e.g.,* nt 994–1003). We used an *in vitro* RNA affinity pulldown technique where biotinylated RNA oligonucleotides serve as ‘bait’ to test whether endogenous proteins from sciatic nerve axoplasm isolates can bind to the RNA bait [24]. Endogenous KHSRP was clearly detected by immunoblotting in affinity pulldowns with the *Prenyl-Cdc42* mRNA nt 764–838 and 801–875 oligonucleotides but not with nt 764–800 or scrambled oligonucleotide affinity pulldowns (Fig 4E). Together, these data show that axonal KHSRP protein can bind to the 3’UTR of *Prenyl-Cdc42* mRNA. However, we were not able to show that axonal KHSRP is bound to endogenous *Prenyl-Cdc42* mRNA using sciatic nerve axoplasm.

### KHSRP deletion increases axonal Prenyl-Cdc42 mRNA

**Fig 5:**
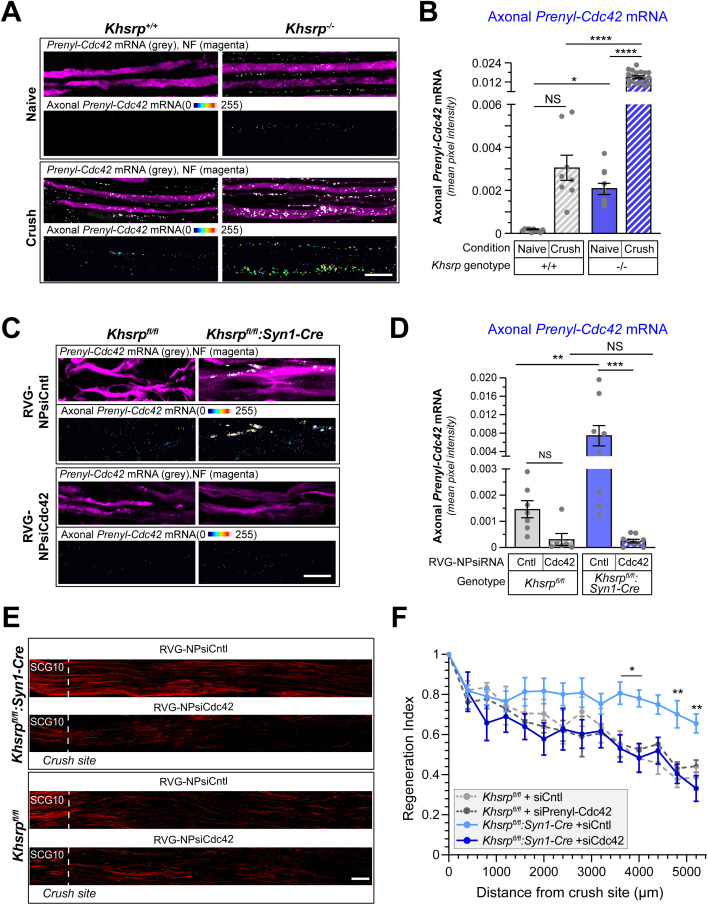
Prenyl-Cdc42 mRNA is increased in KHSRP knockout axons. **A)** Representative smFISH and IF images for naïve and 7 day post-crush injured sciatic nerve from *Khsrp*^*+/+*^ or *Khsrp*^*-/-*^ mice. The upper row of each image set shows the merged confocal *XY* optical plane; the lower row of each image set shows RNA signal overlapping with NF across individual optical planes that was extracted to a separate channel and projected as *XYZ* images; see S5A Fig for representative images of scrambled smFISH probe [Scale bar = 5 µm]. **B)** Quantitation of smFISH signals for RNA probe signals overlapping with NF shown in A as mean ± SEM. See S5B Fig for separate intra-genotype comparison for naïve vs. crush nerves (*N* = 3 biological replicates; NS = not significant, * *P* < 0.05, **** *P* < 0.001 for indicated data pairs by two-way ANOVA with Sidak post-hoc tests for pair-wise comparisons) [Scale bar = 10 µm]. **C)** Representative exposure-matched smFISH/IF for *Cdc42* mRNA in sciatic nerve axons of *Khsrp*^*fl/fl*^:*Syn1-Cre* vs. *Khsrp*^*fl/fl*^ mice at 14 d post-crush shown in **C.** RVG-NPsiRNAs were applied at 7 d post-crush. Merged images show single XY planes and ‘axon only’ images show XYZ project of extracted RNA pixels that overlap with NF in individual Z planes [Scale bar = 5 µm]. **D)** Quantification of axonal *Prenyl-Cdc42* mRNA levels shown in **D.** See S5E Fig for separate intra-genotype comparison for siCntl vs. siCdc42 treated nerves and S5F and S5G Fig for soma RNA values (N = 5 animals per condition; NS = not significant, ** *P* < 0.01, *** *P* < 0.005 for indicated data pairs by two-way mixed effect analysis ANOVA with Sidak post-hoc tests for pair-wise comparisons). **E-F)** Representative images for SCG10 immunostaining (**E**) and regeneration indices (**F**) for *Khsrp*^*fl/fl*^ mice *and Khsrp*^*fl/fl*^:*Syn1-Cre* mice at 14 d post-crush with NP-siRNA injection at 7 d post crush with RVG-NPsiRNAs targeting *Prenyl-Cdc42* mRNA vs. non-targeting siRNA control (N = 5 animals in *Khsrp*^*fl/fl*^:*Syn1-Cre* and N = 3 animals for *Khsrp*^*fl/fl*^ mice; * *P* < 0.05, ** *P* < 0.01 by two-way mixed effect analysis ANOVA with Sidak post-hoc tests for pair-wise comparisons) [Scale bar = 100 µm].

Axonal KHSRP levels increase in rodent sciatic nerve axons after crush injury via localized translation of *Khsrp* mRNA [13]. Since CDC42 protein activity has been shown to increase axon growth [1,4] and the injury-induced increase in axonal KHSRP levels slows regeneration of peripheral nerves [13], we asked whether KHSRP might post-transcriptionally regulate *Prenyl-Cdc42* mRNA within peripheral nerve axons. For this, we compared *Prenyl-Cdc42* mRNA levels in axons of *Khsrp*^*-/-*^ and *Khsrp*^*+/+*^ mice [25] *in vivo* before and 7 days after a sciatic nerve crush injury. In contrast to axons of the cultured DRG neurons used above, axons in the uninjured sciatic nerve are not growing and we previously found that *Prenyl-Cdc42* mRNA only seemed to localize into growing axons of the injured and not the uninjured sciatic nerve in wild type animals [4]. *Prenyl-Cdc42* mRNA was not detected in the uninjured *Khsrp*^*+/+*^ sciatic nerve axons but the mRNA was easily detected in the axons of uninjured *Khsrp*^*-/-*^ sciatic nerves (Figs 5A, 5B, S5A and S5B). 7 day crush injured sciatic nerve axons showed increased axonal *Prenyl-Cdc42* mRNA levels in the *Khsrp*^*+/+*^ mice as anticipated (Figs 5A, 5B and S5B). Remarkably, the regenerating sciatic nerves of the *Khsrp*^*-/-*^ mice showed approximately 5-fold higher axonal *Prenyl-Cdc42* mRNA signals compared to those of the *Khsrp*^*+/+*^ mice (Fig 5B). Thus, KHSRP likely restricts the axonal levels of *Prenyl-Cdc42* mRNA in PNS nerves under both naïve and regenerating conditions.

Both the constitutive *Khsrp*^*-/-*^ mice and *Khsrp*^*fl/fl*^ mice exposed to AAV-Cre show accelerated PNS axon regeneration after traumatic nerve injury [13]. To test for potential functional significance of the elevated *Prenyl-Cdc42* mRNA in *Khsrp*^*-/-*^ axons, we used an *in vivo* siRNA approach to deplete *Prenyl-Cdc42* mRNA from sciatic nerve. We reasoned that delivering an siRNA directly to the sciatic nerve might allow us to preferentially deplete the mRNA from sciatic nerve axons. Thus, we packaged siRNAs targeting *Prenyl-Cdc42* mRNA or non-targeting siRNA (siCdc42 and siCntl, respectively) into a polymersome nanoparticle [26]; the exterior surface of the nanoparticles was tagged with 29 amino acid rabies virus glycoprotein peptide-9R (RVG) that has been that has previously been used to deliver nanoparticle cargos to neurons [27] and has been shown to bind to NCAM [28]. Specificity and efficacy of the *Prenyl-Cdc42* targeting siRNA has been previously published [4]. The siRNA laden RVG-nanoparticles (RVG-NPsiRNA) were initially tested in DRG cultures and showed clear uptake in neuronal soma and axons (S5C Fig). We next tested the RVG-NPsiRNAs *in vivo* by direct injection into the sciatic nerve of wild type mice that had undergone sciatic nerve crush 7 days previously to increase axonal *Prenyl-Cdc42* mRNA. Wild type mice injected with RVG-NPsiCdc42 showed approximately 85% depletion of *Prenyl-Cdc42* mRNA based on RTddPCR analyses of sciatic nerve axoplasm compared to siCntl treated nerves (S5D Fig). We next asked whether *Prenyl-Cdc42* mRNA depletion from axons of KHSRP-deficient mice could decrease the accelerated regeneration seen in mice lacking neuronal KHSRP. For this, we compared sciatic nerve regeneration in *Khsrp*^*fl/fl*^ crossed to *Syn1-Cre* (*KHSRP*^*fl/fl*^:*Syn1-Cre*) vs. *KHSRP*^*fl/fl*^ mice following injection with RVG-NPsiRNAs. *KHSRP*^*fl/fl*^:*Syn1-Cre* mice show altered axon and dendrite growth and nerve injury in adult *Khsrp*^*fl/fl*^ mice where KHSRP was deleted by AAV-Cre show accelerated regeneration [13,29]. Thus, the *KHSRP*^*fl/fl*^:*Syn1-Cre* mice allowed us to focus on effects of elevated axonal *Prenyl-Cdc42* mRNA rather than potential confounding effects of non-neuronal cells in the nerve. 7 days after sciatic nerve crush, RVG-NPsiCdc42 vs. -siCntl were injected proximal to the injury site and at approximately same level in the contralateral (sham) nerve for *KHSRP*^*fl/fl*^:*Syn1-Cre* and *KHSRP*^*fl/fl*^ mice. Axonal *Prenyl-Cdc42* mRNA was depleted by approximately 94% in the RVG-NP-siCdc42 vs. -siCntl injected *KHSRP*^*fl/fl*^:*Syn1-Cre* mice (Figs 5C, 5D and S5E). Murashov et al. (2007) showed that siRNAs can be retrogradely transported in sciatic nerve [30], and recent studies for rats where sciatic nerves were injected with RVG-NPs showed evidence for limited retrograde transport of these particles to the spinal cord [31]. Consistent with this, the L4-5 spinal motor neuron soma for the mice in Fig 4C showed approximately 40% reduction in *Prenyl-Cdc42* mRNA levels by smFISH analysis (S5F and S5G Fig). Thus, although the effect of the RVG-NPsiRNAs is not limited to the site of injection, the axonal *Prenyl-Cdc42* mRNA is overwhelmingly much more depleted in the sciatic nerve axons than in the motor neuron soma that projects axons into the sciatic nerve (i.e., 94 vs. 40% reduction). Nerve regeneration was significantly reduced by *Prenyl-Cdc42* mRNA depletion from the axons of the *Khsrp*^*fl/fl*^:*Syn1-Cre* mice (Fig 5E and 5F). In contrast, depletion of *Prenyl-Cdc42* mRNA had no apparent effect on regeneration in *Khsrp*^*fl/fl*^ mice, which have wild type axonal KHSRP levels (Fig 5E and 5F). Taken together, these findings indicate that stabilization of *Prenyl-Cdc42* mRNA in sciatic nerve axons contributes to the accelerated regeneration that we previously reported in KHSRP knockout mice.

**Fig 6:**
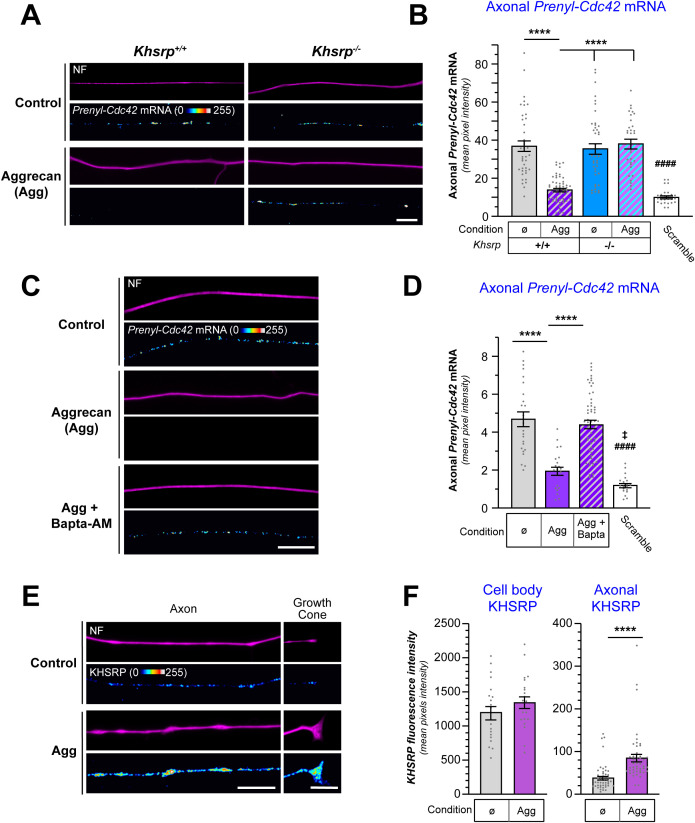
Aggrecan-mediated increase in axonal KHSRP depletes axonal Prenyl-Cdc42 mRNA. **A)** Representative exposure-matched smFISH and IF images for *Prenyl-Cdc42* mRNA and NF in adult mouse *Khsrp*^*+/+*^ or *Khsrp*^*-/-*^ DRG neuron cultures treated with 50 ng/ml aggrecan; see. S6A Fig for representative images of scrambled smFISH probe [Scale bar = 10 µm]. **B)** Quantitation of smFISH signal intensities shown as mean pixel intensity above background ± SEM for axons (*N* ≥ 40 neurons in three independent cultures; **** P* *< 0.001 for indicated data pairs and #### P < 0.001 for scramble vs. all but *Khsrp*^*+/+*^* *+ aggrecan by two-way ANOVA with Sidak post-hoc tests for pair-wise comparisons). **C)** Representative exposure-matched smFISH and IF images for *Prenyl-Cdc42* mRNA plus NF in adult DRG neuron cultures treated with 50 ng/ml aggrecan ± 3 µM BAPTA-AM; see S6B Fig for representative images of scrambled smFISH probe [Scale bar = 10 µm]. **D)** Quantification of smFISH signal intensities shown as mean pixel intensity above background for axons ± SEM (*N* ≥ 20 neurons in three independent cultures; **** P* *< 0.001 for indicated data pairs, ǂ P < 0.05 for scramble vs. aggrecan and #### P < 0.001 for scramble vs. no treatment and Aggrecan + BAPTA by Kruskal-Wallis ANOVA with Dunn post-hoc tests for pair-wise comparisons). **E-F)** Representative exposure-matched IF images for KHSRP protein plus NF in adult DRG neuron cultures ± 50 ng/ml aggrecans are shown in **E.** Quantification of cell body and axonal KHSRP signals from exposure-matched images is shown in F (see S6C Fig for representative IF images for cell bodies; N ≥ 75 neurons over 3 separate cultures; **** P < 0.0001 by student’s t-test) [Scale bars = 10 µm].

Since exposure to the CSPG aggrecan also decreased axonal *Prenyl-Cdc42* mRNA (see Figs 1 and 2), we asked if the decrease in *Prenyl-Cdc42* mRNA after aggrecan exposure is mediated by KHSRP. Axons of *Khsrp*^*+/+*^ neurons showed the anticipated decline in axonal *Prenyl-Cdc42* smFISH signals following aggrecan exposure; however, there was no change in the axonal *Prenyl-Cdc42* mRNA smFISH signals in *Khsrp*^*-/-*^ neurons following aggrecan exposure (Figs 6A, 6B and S6A). Since axonal translation of *Khsrp* mRNA is activated by increased axoplasmic Ca^2+^ [13] and CSPGs are known to increase axonal Ca^2+^ [32,33], we asked if Ca^2+^ is necessary for the aggrecan-induced decrease in axonal *Prenyl-Cdc42* mRNA. Chelation of intracellular Ca^2+^ using BAPTA-AM blocked the aggrecan-induced decrease in axonal *Prenyl-Cdc42* mRNA (Figs 6C, 6D and S6B). Aggrecan treatment also selectively increased axonal and not cell body KHSRP levels (Figs 6E, 6F and S6C). Taken together, these studies indicate that KHSRP binds to a sequence within *Prenyl-Cdc42* mRNA’s nt 801–875, which is functionally distinct from the mRNA’s axonal localization motif (nt 764–800), and Ca^2+^-dependent elevation of axonal KHSRP promotes decay of axonal *Prenyl-Cdc42* mRNA to slow axon growth.

## Discussion

Axonally synthesized proteins have been shown to promote axon growth, axon survival, presynaptic plasticity, and injury signaling [34]. Hundreds to thousands of mRNA are now known to localize into neuronal axons [35]. *Trans-*acting proteins binding to *cis*-elements or motifs within the mRNAs, typically within the mRNA’s UTRs, are responsible for their subcellular localization, but also can impact the translation, storage and stability of those mRNAs once they arrive at their subcellular locale [34]. There have not been any consensus sequence(s) identified that are shared across many axonal mRNAs for individual RNA binding proteins other than the ARE. Though not systematically analyzed in rodents, about 20% of human mRNAs are predicted to have 3’UTR AREs based on sequence analyses [36]. We had previously reported that the 3’UTR of *Prenyl-Cdc42* mRNA is necessary and sufficient for its axonal localization [4] and we show here that the proximal region in *Prenyl-Cdc42* mRNA’s 3’UTR, nt 801–875, is relatively AU-rich, includes a predicted ARE for KHSRP binding, and is bound by KHSRP. This region of *Prenyl-Cdc42* mRNA is greater than 85% conserved at primary sequence level across many vertebrate orthologs. Sequence conservation in UTRs can point to functional regions of an mRNA [18,19], and we find that nt 764–800 and 801–875 constitute two distinct functional regions in *Prenyl-Cdc42* mRNA’s 3’UTR. nt 764–800 promotes axonal localization of *Prenyl-Cdc42* mRNA through an, as yet, unknown *trans*-acting protein(s), while KHSRP binds to nt 801–875 motif and this determines the levels of axonal *Prenyl-Cdc42* mRNA.

ARE-binding has been demonstrated for many different RBPs, including KHSRP, HuC (ELAVL2), HuD (ELAVL4), HuR (ELAVL3), and hnRNPD [36]. Of these, KHSRP and HuD localize to axons and have been suggested to compete for binding to overlapping ARE-containing mRNA populations [37]. For example, the ARE in the 3’UTR of *Gap43* mRNA drives its localization into axons through HuD binding in a complex with Zip Code Binding Protein 1 (ZBP1) [20]. HuD binding also stabilizes *Gap43* mRNA [38], with KHSRP binding through its KH4 domain promotes *Gap43* mRNA’s decay [39]. *Neuritin* (*Nrn1*) mRNA also has a 3’UTR ARE motif that HuD binds in complex with SMN1 protein; this interaction is needed for its localization into axons of cortical but not sensory axons [40,41]. *Nrn1* mRNA localization into sensory axons requires a 5’UTR motif that hnRNP-H1, H2, and F bind [19,41]. *Prenyl-Cdc42* mRNA is similar to *Nrn1* mRNA’s behavior in sensory neurons, in that *Prenyl-Cdc42* mRNA’s localization motif is distinct from its ARE. It is not clear if HuD binds to *Prenyl-Cdc42*’s ARE; though HuD was shown to bind to *Cdc42* mRNA by RIP analyses using cDNA microarrays, that study did not distinguish *Prenyl-* and *Palm-Cdc42* mRNA isoforms [22]. Nonetheless, it is clear that KHSRP binds to *Prenyl-Cdc42*’s sequence within nt 801–875. Also, the remarkable elevation of axonal *Prenyl-Cdc42* mRNA in the *Khsrp*^*-/-*^ mice indicates that KHSRP’s interaction with *Prenyl-Cdc42* mRNA depletes the transcript from distal axons. We previously showed that *Prenyl-Cdc42* mRNA level is very low in uninjured/non-growing axons [4]. The *Khsrp*^*-/-*^ mice showed increased in axonal *Prenyl-Cdc42* mRNA in uninjured conditions indicating that the mRNA can be transported into uninjured axons with basal levels of KHSRP likely limiting the mRNA’s accumulation in distal axons. Thus, KHSRP is likely utilized to dampen axonal synthesis of Prenyl-CDC42 to prevent or attenuate growth of uninjured axons and slow growth of regenerating axons. Consistent with this, depleting *Prenyl-Cdc42* mRNA from PNS axons prevents the accelerated nerve regeneration seen in *Khsrp*^*-/-*^ mice, emphasizing the functional significance of KHSRP’s effect on axonal *Prenyl-Cdc42* mRNA.

KHSRP is a multifunctional RNA binding protein that has been implicated in RNA splicing, RNA transport and decay as well as microRNA biogenesis in addition to promoting ARE-containing mRNA decay [42]. In previous studies, we were not able to show a role for axonal KHSRP in microRNA biogenesis [29]. KSHRP has 4 KH RNA binding domains (KH 1–4), with KH 1–2 domains needed for its RNA splicing function and KH 3–4 needed for its role in RNA decay promotion [21]. Consistent with this, we previously showed that axonal levels of ARE-containing mRNAs are increased when KHSRP’s KH4 is deleted [13,39]. Transcriptome analyses of *Khsrp*^*-/-*^ mouse brain RNA combined with RIP-sequencing for KHSRP’s RNA interactome in wild type mouse brains showed that KHSRP binds to over 400 mRNA targets that increase in brain with loss of *Khsrp* alleles [29]. *Cdc42 effector protein 3* mRNA was shown to increase in *Khsrp*^*-/-*^ mouse brain, but *Cdc42* mRNA levels were not affected in those analyses of whole brain; however, both *Prenyl-Cdc42* and *Palm-Cdc42* mRNA isoforms were identified in KHSRP immunoprecipitates, implying that both isoforms are targets for regulation by KHSRP [29]. The discrepancy between KHSRP binding in wild type mice and lack of *Prenyl-Cdc42* mRNA elevation in cortical brain lysates of *Khsrp*^*-/-*^ mice seen by Olguin et al. may result from the selective interaction of KHSRP with axonal *Prenyl-Cdc42* mRNA [29]. This emphasizes that subcellular RNA-protein interactions and functional effects of those can be missed when looking at whole cell or tissue preparations. A notable limitation of this study is that we have not shown direct binding of axonal KHSRP to endogenous *Prenyl-Cdc42* mRNA; nonetheless, our cumulative data suggest that KHSRP promotes decay of *Prenyl-Cdc42* mRNA locally in axons since KHSRP is introduced into PNS axons through localized translation of its mRNA after axotomy [13].

The outcome of actin filament polymerization by CDC42 activation can be countered by actin filament depolymerization upon RHOA activation [2]. Both CDC42 and RHOA must be activated by GTP binding [2]. Differential regulation of axonal *Prenyl-Cdc42* and *RhoA* mRNA levels and translation in response to the growth-inhibiting CSPG but not the growth-promoting neurotrophin exposure suggest that post-transcriptional regulation of these Rho GTPases can impact axon growth. RHOA activation leads to growth cone collapse and axon retraction and inhibition of the RHOA/ROCK pathway supports axon growth on non-permissive substrates in cultured neurons, including the CSPG used here [43,44]. Traumatic CNS injury such as spinal cord injury (SCI) causes increased levels of growth-inhibiting molecules in the extracellular environment adjacent to the injury, which include CSPGs and myelin proteins [45]. While extent of the contributions of these growth-inhibitory molecules to regeneration failure in the CNS brings some controversy [46], blocking their effects has been proposed as neural repair strategy. RHOA inhibition has been tested pre-clinically and clinically as a therapeutic strategy to overcome the inhibitory environment of the injured CNS. A meta-analysis of experimental SCI models published over 2003–2018 showed that some but not all interventions to inhibit the RHOA pathway promoted *in vivo* axon regeneration [47]. However, local delivery of the RHOA Inhibitor VX-210 did not prove effective for recovery in acute human cervical SCI [48], so it is unclear if other strategies to inhibit the RHOA pathway could bring effective *in vivo* SCI treatment options. Considering that the CSPG aggrecan not only increases axonal RHOA but also depletes *Prenyl-Cdc42* mRNA from axons, our data raise the possibility that inhibition/inactivation of the RHOA pathway still leaves the axon in a low growth state since this would not prevent the depletion of *Prenyl-Cdc42* mRNA from axons in the injured CNS. Indeed, strategies that increase axonal CDC42 activity may be needed to effectively promote axon regeneration in the non-permissive environment of the injured CNS. It should be noted that our data do not exclude the possibility of axonal transport for soma-synthesized RHOA and Prenyl-CDC42 proteins; however, our data clearly show axonal RNA and translation for these proteins are differentially regulated by these growth modulating stimuli.

CSPGs binding to the transmembrane receptors PTPσ and LAR activates RHOA/ROCK signaling to inhibit axon growth [49]. *RhoA* mRNA was previously been shown to localize into axons [6], and its local translation was subsequently shown to be increased by CSPGs [7]. CSPG treatment increases intra-axonal Ca^2+^ in cultured DRG neurons [33]. Translation of some mRNAs, including axonal *Khsrp* mRNA [13], is increased by Ca^2+^-dependent activation of PERK and subsequent phosphorylation of eIF2α [50]. Thus, a CSPG-driven increase in axonal Ca^2+^ could indeed increase local *Khsrp* mRNA translation to subsequently deplete *Prenyl-Cdc42* mRNA from axons. Consistent with this, the CSPG-dependent depletion of *Prenyl-Cdc42* mRNA from axons was attenuated by chelating intra-cellular Ca^2+^ with BAPTA-AM. CSPGs as well as the CNS axon growth-inhibiting myelin-associated glycoprotein (MAG) attenuate axonal transport of mitochondria through a mechanism requiring elevation of axonal Ca^2+^ and activation of RHOA [32]. This raises the possibility that signals from other CNS growth-inhibitory molecules similarly bring a dual hit to block axon regeneration by decreasing Prenyl-CDC42 synthesis and increasing RHOA synthesis in distal axons. Given the reciprocal regulation of *RhoA* and *Prenyl-Cdc42* mRNAs by CSPGs and the increased axonal transport and translation of *Prenyl-Cdc42* mRNA in response to neurotrophins, optimal growth of injured axons in the CNS may require simultaneously inhibiting the RHOA pathway and increasing axonal translation of *Prenyl-Cdc42* mRNA.

## Materials and methods

### Ethics statement

All animal work was approved by the Institutional Animal Care and Use Committee at the University of South Carolina (AUP 2633-101765-012023). Recombinant DNA work was approved by the Institutional Biosafety Committee at the University of South Carolina (Protocol # 1-0114-0425).

### Key reagents and resources

S1 Table contains details for key resources and source for those resources that were used in this study.

### Animal care and use

Institutional Animal Care and Use Committee of the University of South Carolina approved all animal procedures. Sprague Dawley rats (175–250 g) were used for preparing sciatic nerve axoplasm. Male and female wild type C57Bl/6 (*Khsrp*^*+/+*^), constitutive *Khsrp* knockout (*Khsrp*^*-/-*^) [25], and conditional *Khsrp*^*fl/fl*^ [29] mice were used for sciatic nerve injury and DRG culture experiments as indicated in the results. For neuronal specific *Khsrp* knockout, male *Khsrp*^*fl/fl*^ mice were crossed to female B6.Cg-Tg(Syn1-cre)671Jxm/J (*Syn1-Cre*; Jackson Laboratories) mice. All animals were euthanized by CO_2_ asphyxiation per IACUC guidelines.

For nerve crush surgery, animals were anesthetized with isoflurane by inhalation (5% induction and 2% maintenance). Anesthetized animals were subjected to sciatic nerve crush at mid-thigh level as previously described [51]. Briefly, the nerve was exposed by blunt dissection and then crushed with # 2 fine jeweler’s forceps, twice for 15 sec each; success of the axotomy was monitored by the initial contraction of the hind limb upon applying pressure to the nerve and then lack of hind paw extension during and upon recovery from anesthesia.

For *in vivo* RNA depletion from sciatic nerve axons, polymersomes with siRNAs (see below) were delivered by injecting 6 µl of polymersome solution in 1x PBS that contained an equivalent of 40 nM siRNAs. Polymersomes were injected into the sciatic nerve of anesthetized mice (see above) at 7 days following nerve crush injury (performed as above) just proximal to the injury site. Delivery of polymersomes was confirmed by RTddPCR for *Prenyl-Cdc42* and *Gapdh* mRNAs, visualization of the polymersomes’ fluorophore in the nerve, and smFISH/IF for *Prenyl-Cdc42* mRNA and neurofilament protein.

### Mouse genotyping

Genotyping for constitutive KHSRP knockout was performed using PCR with primers spanning the exon 1 to exon 13 deletion of the mouse *KHSRP* gene or wild type sequence as previously described [29]. For this, DNA was extracted from ear punches taken at weaning. Primers used for genotyping are as follows (5’ to 3’): Khsrp forward P1 – TTCCGAAGCTCTGACTGGTC, Khsrp reverse P2 – CGGTGTTGTAGTCCGACATG, and Khsrp reverse P3 – AAGGGTCCAGGGTTGAAAGG. PCR products were analyzed by agarose gel electrophoresis with SYBRSafe DNA Gel Stain (ThermoFisher).

*Khsrp*^*fl/fl*^ mice were generated by Biocytogen using CRISPR/EGE-based gene editing to insert loxP sites between exons 1 and 2 and exons 6 and 7 as described [13]. Genotyping for loxP insertion was performed using following primers (5’ to 3’): 5’ LoxP forward – AGTGTTATGTGCTGGTGTGACCTGG, 5’ LoxP reverse – GTGCTTACCCTTGACAGGGAGTGTC, 3’ LoxP forward – CTATGGTGTCACCTCTCAGTGCTGC, and 3’ LoxP reverse – CACGTAGAGGCCAAAGCAAGAGGAC. PCR products were analyzed by agarose gel electrophoresis with SYBRSafe DNA Gel Stain (ThermoFisher). For specific Cre expression in neuronal cells, *Khsrp*^*fl/fl*^ mice were crossed with Syn1-Cre mice [29]. The following primers were used to detect Syn1-Cre-mediated recombination (5’ to 3’): forward transgene – CTCAGCGCTGCCTCAGTCT, reverse transgene – GCATCGACCGGTAATGCA, forward IPC – CAAATGTTGCTTGTCTGGTG, and reverse IPC – GTCAGTCGAGTGCACAGTTT. PCR products were analyzed by agarose gel electrophoresis with SYBRSafe DNA Gel Stain (ThermoFisher).

### Primary neuron culture

Dissociated cultures of adult DRGs were prepared as described (Twiss et al., 2000). DRGs were harvested in Hybernate-A medium (BrainBits) and then dissociated with 2,000 units/ml Collagenase type 2 (ThermoFisher) at 37°C, 5% CO_2_ for 15 min. Ganglia were triturated using a fire polished Pasteur pipet, diluted into 9 volumes DMEM/F12 (ThermoFisher), and then pelleted at 100 xg for 5 min. After pelleting, dissociated ganglia were washed in DMEM/F12 and then cultured in DMEM/F12, 1 x N1 supplement (Sigma-Aldrich), 10% fetal bovine serum (Hyclone), and 10 µM cytosine arabinoside (Sigma-Aldrich) and plated onto poly-L-lysine (Sigma-Aldrich) and laminin (ThermoFisher)-coated substrates.

For transfections, dissociated ganglia were pelleted at 100 x g for 5 min and resuspended in 100 µl ‘Nucleofector solution’ (Rat Neuron Nucleofector kit; Lonza). 4–6 µg of each plasmid was electroporated using the AMAXA Nucleofector device (G013 program; Lonza) before plating. Dissociated ganglia were then plated as above and analyzed 48–72 h later.

For RVG-NP treatments, dissociated cultures were exposed to RVG-NPsiRNAs (see below) for 2 hours, fixed in buffered 4% PFA, and then directly imaged by confocal microscopy.

### Plasmid constructs

GFP^MYR^ translation reporter originally provided by Dr. Erin Schuman (Max-Plank Inst., Frankfurt) [16]. Mammalian expression plasmids with the coding sequence of the GFP^MYR^ containing cDNA corresponding to the 5’ and 3’UTRs of rat *Prenyl-Cdc42* (GenBank Accession # XM_008764286; Lee et al., 2021) were used as a basis for 3’UTR deletion constructs GFP^MYR^3’prenyl-Cdc42^764-913^ and GFP^MYR^3’prenyl-Cdc42^914-2164^. Constructs were produced by digestion with either Not1 and BstX1 for GFP^MYR^3’prenyl-Cdc42^764-91*3*^, or Bstx1 and EcoR1 for GFP^MYR^3’prenyl-Cdc42^914-2164^. 3’ overhangs were then filled using Klenow fragment (New England Biolabs) and re-ligated.

To create expression constructs for deletions of the Cdc42 3’UTR nt 764–913, double stranded oligonucleotides corresponding to nt 764–800, 764–838, 801–875, and 839–913 of rat *Prenyl-Cdc42* mRNA were custom synthesized by Integrated DNA Technologies (IDT). These 3’UTR segments were engineered with 5’ Not1 and 3’ Xho1 restriction sites and used to replace the 3’UTR in GFP^MYR^5’CamK2α/3’Actg plasmid. This plasmid contains the 5’UTR of calcium/calmodulin dependent protein kinase II alpha (CamK2α) that has previously been shown to lack any activity for axonal localization [52].

GFP^MYR^5’/3’prenyl-Cdc42 used in our FRAP analyses was previously generated in our lab (Lee et al., 2021). mCherry^MYR^5’/3’RhoA was generated by replacing the 5’ and 3’UTR of mCherry^MYR^5′/3′Kpnb1 [13] with PCR generated sequences. Primers used for cloning are as follows (5’ to 3’): *RhoA* 5’UTR *HinD3* forward – CCCAAGCTTTGAGTATAAAATAGCAACTCGGTCTTTTATAG, *RhoA* 5’UTR *BamH1* reverse – CGGGATCCCACTTATGAAGGTGCTGAAGAAACTC, *RhoA* 3’UTR *Not1* forward – GGGGCGGCCGCAGCCTTGTGAC, *RhoA* 3’UTR *Xho1* reverse – GGGCTCGAGTTTAGAAAACTGCCT, corresponding to rat *RhoA* (GenBank Accession # XM_006243699). The 5’UTR was engineered with 5’ Hind3 and 3’ BamH1 restriction sites and the 3’ UTR was engineered with 5’ Not1 and 3’ Xho1 restriction sites and used to replace the 5’ and 3’ UTRs of mCherry^MYR^5’/3’Kpnb1.

### Polymersome delivery of siRNAs

Synthetic siRNAs were purchased from Dharmacon (Horizon Discovery Biosciences). The *Prenyl-Cdc42* and non-targeting control siRNA sequences were previously published [4]. siRNAs were packaged into polymeric nanoparticles called polymersomes that were labeled with a peptide of rabies virus glycoprotein, RVG29 (called RVG herein). Polymersomes were made from solvent injection of a 50:50 mixture of block co-polymer polyethylene glycol (PEG, 1000 kDa)-b-polylactic acid (PLA, 5000 kDa) and PEG(1000)-b-PLA(5000)-maleimide, then lyophilized prior to siRNA encapsulation. Cysteine conjugated RVG is added to solution, enabling a thiol coupling reaction on the polymersome surface to attach the RVG. RVG attachment is confirmed via a shift in surface charge from negative to positive via zeta potential measurements [26]. RVG-tagged polymersomes co-encapsulated 13.6 ± 4.6 µg siRNA/mg polymer with 53 ± 8 µg membranous DiD/mg polymer. RVG-tagged siRNA loaded polymersomes were concentrated to 100 mM prior to injection.

siRNAs were first tested for uptake in primary mouse dissociated DRG cultures based on DiD fluorescence emission and sciatic nerve in vivo by RTddPCR for *Prenyl-Cdc42* vs. *Gapdh* mRNA (see below).

### Fluorescence in situ hybridization and immunofluorescence

Custom Stellaris oligonucleotide probes with 5′ Quasar 570 or 670 labels (BioSearch Tech.; see S1 Table) were used for smFISH combined with IF to detect *Prenyl-Cdc42, RhoA* and *GFP* mRNAs and Neurofilament (NF). Scrambled probes were used as control for specificity. For cultured neurons, coverslips with dissociated DRGs were briefly rinsed in phosphate buffered saline (PBS), fixed for 15 min in 2% PFA in PBS, and then processed for pre-hybridization and hybridization as described [53]. Primary antibodies to detect neurofilament (NF) consisted of a cocktail of mouse RT97 (1:500, Devel. Studies Hybridoma Bank) and SMI312 (1:250; BioLegend). FITC-conjugated donkey anti-mouse (1:200, Jackson ImmunoRes.) was used as the secondary antibody.

smFISH/IF was performed on sciatic nerve and spinal cord cryosections as described previously [53]. Briefly, tissues were immersion fixed overnight in 2% PFA in PBS at 4°C and then cryoprotected overnight in 30% sucrose in 1x PBS at 4°C. Samples were processed for cryosectioning (10 µm thickness), sections were adhered to Superfrost^plus^ glass slides (Fisher) and then stored at -20°C until use. Slides were dried at 37°C for 1 h and then brought to room temperature. All subsequent steps performed at room temperature unless indicated otherwise. Sections were washed for 10 min in PBS, then 10 min in 20 mM glycine three times followed by three 5 min incubations in fresh 0.25 M NaBH_4_. Sections were rinsed in 0.1 M triethanolamine (TEA), incubated for 10 min in 0.25% acetic anhydride in 0.1 M TEA, and washed twice with 2x saline-sodium citrate (SSC) buffer. Sections were then dehydrated through graded ethanol solutions (70, 95, and 100% for 3 min each) followed by delipidation in chloroform for 5 min. Sections were rehydrated in 100 and 95% ethanol for 3 min each, equilibrated in 2x SSC and then incubated at 37°C in a humidified chamber in hybridization buffer (50% dextran sulphate, 10 μg/ml E. coli tRNA, 10 mM ribonucleoside vanadyl complex [Millipore Sigma], 80 μg BSA, and 10% formamide in 2 × SSC) for 5 min. Hybridization and immunolabeling were performed overnight in a humidified chamber in hybridization buffer containing 7 µM of each probe. Sections were then washed twice in 2x SSC plus 10% formamide at 37°C for 30 min and once in 2x SSC for 5 min. After permeabilization in PBS plus 1% Triton X-100 (PBST) for 5 min, nerve sections were incubated for 1 h in a cocktail of mouse RT97 (1:500, Devel. Studies Hybridoma Bank) and SMI312 (1:250; BioLegend) primary antibodies. FITC-conjugated donkey anti-mouse-IgG antibody (1:200) in 0.3% Triton X-100 supplemented with 1 × blocking buffer (Roche). Spinal cord samples were incubated in 1:400 dilution fluorescent Neurotrace 640/660 (ThermoFisher) for 1 h after FISH. After washing in PBS for 5 min, sections were post-fixed in 2% PFA in PBS for 15 min [54], washed in PBS three times for 5 min, and then rinsed in DEPC-treated water. Both the cultured neurons and tissues were mounted using Prolong Gold Antifade (Invitrogen).

All smFISH/IF performed on tissue sections was imaged by confocal microscopy using a Leica SP8X or Leica Stellaris confocal microscopes with HyD detectors and matched post-processing measures to distinguish axonal signals from non-neuronal signals. Scrambled probes were used to assign maximum acquisition parameters to limit any nonspecific signal from the probes.

Standard IF was performed as previously described [41] with all steps at room temperature unless specified otherwise. Cultures were fixed with 4% PFA in PBS for 15 min and washed 3 times in PBS. PBS washed cultures were permeabilized with 0.3% Triton X-100 in PBS for 15 min and then blocked in PBST plus 5% BSA for 1 h. Tissues were immersion fixed in 4% PFA in PBS for 4 h, cryoprotected overnight in 30% sucrose at 4°C, and then processed for cryosectioning at 25 µm thickness. Sections were stored at -80°C and then warmed to RT, and permeabilized for 15 min in 0.3% Triton X-100. Sections were washed in PBS wash 3 times and then blocked for 1 h in PBST containing 10% normal donkey serum. Tissue and culture samples were incubated with primary antibodies overnight at 4°C. Primary antibodies for cultured neurons consisted of rabbit anti-CDC42 (1:500; Abcam), mouse anti-RhoA (1:25; Abcam), and chicken anti-NF (1:500, NFM, NFL and NFH; Aves Labs). Primary antibodies for tissues consisted of rabbit anti-SCG10 (1:100; Novus Biologicals). After washing in PBS, coverslips were incubated with combination of FITC-conjugated donkey anti-chicken and Cy5-conjugated donkey anti-rabbit or anti-chicken (all at 1:500; Jackson ImmunoRes.) as secondary antibodies for 1 h. After 1 h, samples were washed 3 times in PBS, rinsed with distilled H_2_O, and mounted with Prolong Gold Antifade with DAPI.

### Fluorescence recovery after photobleaching

FRAP analyses were performed as published [18], with minor modifications. DRG neurons were transfected with GFP^MYR^5’/3’prenyl-Cdc42 and mCherry^MYR^5’/3’RhoA as above. Cells were maintained at 37°C, 5% CO_2_ during imaging. 488 nm and 587 nm laser lines on a Leica SP8X confocal microscope with HyD detectors were used to bleach GFP and mCherry signals, respectively (argon laser at 70% power, pulsed every 0.82 sec for 80 frames). Leica 63x/1.4 NA oil immersion objective was used with the confocal pinhole set to 3 Airy units to ensure full thickness bleaching and acquisition (Yudin et al., 2008). 488 nm and 587 nm laser lines on the Leica SP8X confocal microscope with HyD detectors were used to bleach GFP and mCherry signals, respectively (argon laser at 70% power, pulsed every 0.82 sec for 80 frames). Regions of interest (ROI) for bleaching and analyses consisted of at least 50 µm of terminal axon length separated from the soma by at least 250 µm. Prior to photobleaching, two frames were acquired at 60 sec intervals to determine baseline fluorescence in the ROI (15% power with pulsed white light laser; 498–530 nm for GFP; 597–630 nm for mCherry). The same excitation and emission parameters were used to assess recovery over 15 min post-bleach with images acquired every 30 sec. For some experiments, 50 ng/ml of aggrecan (Sigma-Aldrich) or a neurotrophin cocktail consisting of 10 ng/ml each of NT3 (Alamone Labs) + BDNF (Alamone Labs) + NGF (Inotiv) was bath applied immediately prior to imaging. To test whether any fluorescence recovery in axons was due to translation, DRG cultures were treated with 100 µM anisomycin (Sigma) for 30 min prior to photobleaching.

### RNA isolation and analyses

RNA was isolated from mouse DRG culture lysates and affinity pull down samples using the RNeasy Microisolation kit (Qiagen). Fluorimetry with Ribogreen (Invitrogen) was used for RNA quantification for total RNA isolates. For analyses of total RNA levels and inputs from immunoprecipitates, RNA yields were normalized for mass across samples prior to reverse transcription (RT) using Superscript IV Vilo (ThermoFisher). For co-immunoprecipitation of RNA, samples were processed based on equivalent proportions of the precipitate rather than normalizing to RNA mass. Droplet digital PCR (ddPCR) was performed using Taqman probe sets (IDT) and QX200 droplet reader (Bio-Rad). Primer/probe sets were as follows (5’ to 3’): prenyl-Cdc42 sense primer – CGTTTGTGGGGATTTGCGTT, prenyl-Cdc42 antisense primer – GACAGACGACCTGCACCTAC, prenyl-Cdc42 Probe -/56-FAM/GCCCCCTTG/ZEN/CCCTTCCGGTA/3IABkFQ/, GFP sense primer – CTGCTGCCCGACAACCAC, GFP antisense primer – TCACGAACTCCAGCAGGAC, and GFP probe -/56-FAM/CCAGTCCGC/ZEN/CCTGAGCAAAGACC/3IABkFQ/.

### Affinity isolation of RNA-interacting proteins

RNA-Protein pull-down was performed as described [24]. We used axoplasm from sciatic nerve as a source of proteins. To obtain enriched axonal contents, approximately 3 cm segments of rat sciatic nerve were dissected and axoplasm was extruded into 20 mM HEPES [pH 7.3], 110 mM potassium acetate, and 5 mM magnesium acetate (‘nuclear transport buffer’) supplemented with 1x protease/phosphatase inhibitor cocktail (Roche) and 40 U/µl RNasin Plus (Promega) as previously described [55]. After clearing by centrifugation at 20,000 xg at 4°C for 30 min, supernatants were mixed with 5’ biotin-conjugated RNA oligonucleotides (IDT), which had been adsorbed to streptavidin (SA) dynabeads (ThermoFisher) [19], and incubated for 4 h at 4˚C. Beads were precipitated using a magnetic rack and then washed extensively with 10 mM HEPES (pH 7.4), 3 mM MgCl_2_, 250 mM NaCl, 1 mM DTT, and 5% glycerol. Bound proteins were eluted by treating with 50 µg/ml RNase A (Sigma-Aldrich) in wash buffer for 15 min at 37˚C [19]. Proteins were denatured by boiling at 95˚C in Laemmli sample buffer for 5 min, fractioned by SDS/PAGE, and then transferred to nitrocellulose membranes for Immunoblotting.

### RNA co-immunoprecipitation

For co-precipitating RNAs with proteins, DRG cultures were lysed in 100 mM KCl, 5 mM MgCl_2_, 10 mM HEPES [pH 7.4], 1 mM DTT, and 0.5% NP-40 (‘RIP buffer’) supplemented with 1x protease inhibitor cocktail and RNasin Plus. Lysates were passed through 25 Ga needle 5–7 times and then cleared by centrifugation at 12,000 xg for 20 min. Cleared lysates were incubated with Protein G-Dynabeads (ThermoFisher) for 30 min to reduce non-specific binding. After collection, supernatants were then incubated with rabbit anti-KHSRP (5 μg, Novus) or rabbit IgG (5 μg, Jackson ImmunoRes.) for 3 h at 4˚C with rotation. Immunocomplexes were incubated with Protein G-Dynabeads for an additional 2 h at 4°C with rotation. Beads were washed six times with cold RIP buffer. An aliquot was reserved for validating protein precipitation (see below), and bound RNAs were isolated by addition of RNeasy Microisolation kit lysis buffer and analyzed by RTddPCR (see above).

For validation of pull-down of protein, the reserved aliquot from the immunoprecipitates was resuspended in 1 x Laemmli sample buffer and denatured by boiling at 95°C x 5 min. Supernatants were then processed for immunoblotting.

### Protein electrophoresis and immunoblotting

Protein concentrations were determined by BCA assay. Cell lysates and axoplasm were normalized for concentration prior to electrophoresis or immunoprecipitation. Lysates, immunoprecipitates, and RNA affinity isolates were denatured by boiling in Laemmli sample buffer, fractionated by SDS-PAGE, and electrophoretically transferred to nitrocellulose membranes. Blots were incubated for 1 h at room temperature in blocking buffer (5% non-fat dry milk in Tris-buffered saline with 0.1% Tween 20 [TBST]). Membranes were incubated overnight incubation at 4°C with rocking in mouse anti-KHSRP (1:1000; Abcam) diluted in TBST plus 5% BSA. After washing in TBST, blots were incubated HRP-conjugated anti-mouse IgG antibodies (1:5000; Jackson ImmunoRes.) diluted in blocking buffer for 1 h at room temperature. After washing in TBST (3 times), signals were detected using Clarity Western ECL Substrate (Bio-Rad).

### Quantitation and statistical details

All imaging experiments included at least three technical replicates for each culture, and each experiment was replicated across at least three separate culture preparations. For molecular studies using transfected cultures, analyses were performed across at least three separate culture preparations.

For smFISH on tissue sections the Z stacks of XY optical planes were captured at two locations along each nerve section. The *Colocalization Plug-in* for *NIH ImageJ* (https://imagej.nih.gov/ij/ plugins/colocalization.html) was used to extract RNA signals from smFISH probes in each optical plane that overlapped with NF signals as an ‘axon only’ mRNA signal. All smFISH signal quantifications for axonal mRNA signals from tissue sections were generated by analysis of pixel intensity across each XY plane of the extracted ‘axon only’ channels for the image sequences using *ImageJ*. These smFISH signal intensities across the individual XY planes were then normalized to the area of NF immunoreactivity in each XY plane and averaged across the image stack [53]. The relative mRNA signal intensity was then determined from the average in each biological replicate.

For FRAP assays, fluorescent intensities in the bleached region of interest (ROI) were calculated using Leica LASX software. For normalizing across experiments, fluorescence intensity value for the ROI at t = 0 min post-bleach from each image sequence was set as 0%. The relative fluorescence recovery at each time point after photobleaching was then calculated by normalizing to the pre-bleach fluorescence intensity of the ROI (set at 100%). All bleached ROIs were at least 250 µm from the cell soma, so any significant fluorescence recovery occurring in less than 15 min post-bleach interval can be attributed to local protein synthesis rather than anterograde transport of reporter protein into the ROI if recovery was significantly attenuated by protein synthesis inhibitors.

Quantitative data are reported as mean ± SEM. GraphPad Prism 9 software was used for all statistical analyses. Data outliers were removed using Prism’s ROUT with Q set at 1% (*i.e.*, maximum allowable false discovery rate). Normality of data sets was assessed, if all samples pass normality test for Gaussian distribution, an ordinary ANOVA was performed with Tukey multiple comparisons test. Data that failed Gaussian distribution were then analyzed as nonparametric distribution with a Kruskal-Wallis test with Dunn’s multiple comparisons test. Statistical comparisons across experimental conditions in addition to genotype were analyzed either by two-way repeated measures ANOVA with Tukey post-hoc tests for pair-wise comparisons for data sets with equal numbers per group or two-way mixed effect analysis ANOVA with Sidak post-hoc tests for pair-wise comparisons for data sets with unequal number in each group. Pairwise comparisons were performed by either Student or Welch’s t-test as indicated.

## Supporting information

S1 Table Key Reagents and Resources. (DOCX)

S1 Fig Differential regulation of axonal Prenyl-Cdc42 and RhoA mRNA levels (accompanying Fig 1). **A)** Representative IF images with no primary antibody as negative control for Fig 1D (see Fig 1E-F for quantifications). **B)** Quantitation of CDC42 signal intensities shown as mean pixel intensity above background ± SEM for cell bodies (*N *≥ 15 neurons in three independent cultures; NS = not significant between indicated data pairs by ordinary one-way ANOVA with pair-wise comparison with Tukey post-hoc tests). **C)** Quantitation of RHOA signal intensities shown as mean pixel intensity above background ± SEM for cell bodies (*N *≥ 15 neurons in three independent cultures; NS = not significant, **** P < 0.001 between indicated data pairs by Kruskal-Wallis ANOVA with pair-wise comparison with Dunn post-hoc tests).
(TIF)

S2 Fig Differential translation of axonal Prenyl-Cdc42 and RhoA mRNA (accompanying Fig 2). A-B) Representative FRAP image sequences for DRG neurons co-transfected with GFP^MYR^5’/3’prenyl-Cdc42 (A), and mCherry^MYR^5’/3’RhoA (B) at 72 h post-transfection are shown. Boxed regions represent the photobleached ROIs (see quantification in Fig 2B-C) [Scale bar = 20 µm].
(TIF)

S3 Fig Sequence alignment for vertebrate Prenyl-Cdc42 mRNA orthologs (accompanying Fig 3). *Clustal Omega* multiple sequence alignment [55] for the 3’UTR of *Prenyl-Cdc42* mRNAs are shown. Blue boxed regions show nucleotide conservation across orthologs. Nucleotide numbers labelled above start at the first nucleotide of the 3’UTR for *Xenopus tropicalis*. Beneath are graphical representations of consensus (% identity) and occupancy as well as a consensus aligned sequence.
(TIF)

S4 Fig Cell body expression of GFP^MYR^3’prenyl-cdc42 mRNAs (accompanying Figs 3–4). **A)** Representative smFISH images for scramble FISH probe as negative control exposure matched to those in Fig 3B & 3D (see Fig 3C & 3E for quantitative data) [Scale bar = 10 µm]. **B-C)** Quantitation of smFISH signal intensities shown as mean pixel intensity above background ± SEM for cell bodies corresponding to Figu 3D-E (*N *≥ 40 neurons in three independent cultures; not significant between any data pairs by one-way ANOVA, pair-wise comparison with Tukey post-hoc tests).
(TIF)

S5 Fig KHSRP regulates axonal Prenyl-Cdc42 mRNA levels (accompanying Fig 5). **A)** Representative smFISH images for 7 day post-crush injured sciatic nerve showing signals for scramble probe exposure matched to Fig 5A (see Fig 5B for quantitation) [Scale bar = 5 µm]. **B)** Quantitation of smFISH signals for RNA probe signals for individual *Khsrp* genotypes from Fig 5B as mean ± SEM (*N *= 3 biological replicates; **** P < 0.001 by Welch’s t-test for individual comparisons). **C)** Representative transmitted light image merged with signals for Alexa488-labeled RGV-NP-siCdc42 (Green) treated wild type mouse DRG cultures. Both the soma (left) and distal axon with growth cone (right) show apparent intracellular DiD signals (arrows) [Scale bar right panel = 25 µm, left panel = 10 µm]. **D)** Quantification of axoplasm *Prenyl-Cdc42* and *Gapdh* mRNA levels from wild type mice injected with RVG-NP-shCntl vs -siCdc42 (N = 3 animals per condition; **** P < 0.001 by Welch’s t-test for individual comparisons). **E)** Quantification of smFISH for axonal *Prenyl-Cdc42* mRNA levels from Fig 5B separated as *Khsrp*^*fl/fl*^ and *Khsrp*^*fl/fl*^:*Syn1-Cre* mice treated with RVG-NP-siCntl vs. -siCdc42 as mean ± SEM (N = 3–5 animals per condition; * *P *< 0.01 by Welch’s t-test for individual comparisons). **F-G)** Representative exposure matched smFISH images for *Prenyl-Cdc42* mRNA + Nissl substance (**F**) and motor neuron smFISH signal quantitation (**G**) for *Khsrp*^*fl/fl*^:*Syn1-Cre* mice that had received RVG-NP-siCntl vs. -siCdc42 nerve injections 5 days prior to euthanasia (*N *≥ 40 neurons, N = 3 animals; ** P < 0.01 and **** P < 0.0001 between indicated data pairs by Kruskal-Wallis ANOVA with pair-wise comparison with Dunn post-hoc tests) [Scale bar = 10 µm].
(TIF)

S6 Fig Aggrecan triggered increase in KHSRP is limited to axons (accompanying Fig 6). **A)** Representative smFISH images using scramble probe as negative control in adult mouse *Khsrp*^*-/-*^ DRG neuron cultures exposure matched to Fig 6A (see Fig 6B for quantitation) [Scale bar = 10 µm] **B)** Representative smFISH images using scramble probe as negative control in adult mouse DRG neuron cultures exposure matched to Fig 6C (see Fig 6D for quantitation) [Scale bar = 10 µm]. **C)** Representative exposure matched IF images for KHSRP protein in cell bodies of cultured mouse DRG neurons exposed to aggrecan as in Fig 6E (see Fig 6F for quantitation) [Scale bar = 25 µm].
(TIF)
