# Supplementary material for: KHSRP-mediated decay of axonally localized prenyl-Cdc42 mRNA slows nerve regeneration
Source: PLoS Genet. 2025 Nov 7;21(11):e1011916. doi: 10.1371/journal.pgen.1011916 (PMC12614808; doi:10.1371/journal.pgen.1011916)
Supplement: S1 Table — (DOCX) [file pgen.1011916.s001.docx]

**S1 TABLE: *Key resources used.***

| **REAGENT or RESOURCE** | **SOURCE** | **IDENTIFIER** |
| --- | --- | --- |
| ***Antibodies*** | | |
| RT97 mouse anti-neurofilament | Devel. Studies Hybridoma Bank | Cat # RT97;  RID: AB_528399 |
| SMI312 mouse anti-NF | BioLegend | Cat # 837904;  RRID: AB_2566782 |
| Rabit anti-SCG10 (Stathmin 2) | Novus Biologicals | NBP1-49461 |
| FITC-conjugated donkey anti-mouse | Jackson Immuno. | Cat # 715-095-150; RRID: AB_2340792 |
| Rabbit anti-CDC42 | Abcam | Cat # ab187643;  RRID: AB_2818943 |
| Mouse anti-RhoA | Abcam | Cat # ab54835;  RRID: AB_945224 |
| Rabbit anti-KHSRP | Novus | Cat # NBP1-18910;  RRID: AB_2130505 |
| Mouse anti-KHSRP | Abcam | Cat # ab56438; RRID: AB_943921 |
| Chicken anti-Neurofilament (heavy) | Aves labs | Cat # NFH;  RRID: AB_2313552 |
| Chicken anti-Neurofilament (medium) | Aves labs | Cat # NFM;  RRID: AB_2313554 |
| Chicken anti-Neurofilament (light) | Aves labs | Cat # NFL;  RRID: AB_2313553 |
| FITC-conjugated donkey anti-chicken | Jackson Immunoresearch | Cat # 703-095-155;  RRID: AB_2340356 |
| Cy5-conjugated donkey anti-rabbit | Jackson Immunoresearch | Cat# 711-095-152; RRID: AB_2315776 |
| Cy5-conjugated donkey anti-chicken | Jackson Immunoresearch | Cat # 703-175-155  RRID: AB_2340365 |
| HRP-conjugated donkey anti-mouse (H+L) ML | Jackson Immunoresearch | Cat # 715-005-150  RRID: AB_2340758 |
| Rabbit IgG | Jackson Immunoresearch | Cat # 011-000-002  RRID:  AB_2337117 |
| Protein G Dynabeads | Thermofisher | Cat # 10003D |
| Streptavidin Dynabeads | Thermofisher | Cat # 65305 |
| ***Biological samples*** |  |  |
| Fetal bovine serum | Hyclone | Cat # SH30071 |
| ***Chemicals, peptides, and recombinant proteins*** | | |
| NT3 | Alomone Labs | Cat # N-260 |
| BDNF | Alomone Labs | Cat # B-250 |
| NGF | Inotiv (Harlan Labs) | Harlan Cat # 5017 |
| Aggrecan | Sigma-Aldrich | Cat # A1960 |
| Anisomycin | Sigma-Aldrich | Cat # A5862 |
| BAPTA-AM | Millipore-Sigma | Cat # 196419 |
| Protease inhibitor cocktail | Roche | Cat # 04693132001 |
| DMEM/F12 | Thermofisher | Cat # 11320033 |
| Hybernate A medium | BrainBits | Cat # SKU HA |
| N1 medium supplement | Sigma-Aldrich | Cat # N6530 |
| Collagenase type 2 | Thermofisher | Cat # 17101015 |
| Cytosine arabinoside (Ara-C) | Sigma-Aldrich | Cat # C6645 |
| Poly-L-Lysine | Sigma-Aldrich | Cat # P4707 |
| Laminin | Thermofisher | Cat # 23017015 |
| Rat Neuron Nucleofector kit | Lonza | Cat # VPG-1003 |
| Clarity^TM^ Western ECL Substrate | Bio-Rad | Cat # 1706060 |
| SyproRuby protein stain | Thermofisher | Cat # S12000 |
| Prolong Gold Antifade mounting medium | Thermofisher | Cat # P36934 |
| Prolong Gold Antifade mounting medium with DAPI | Thermofisher | Cat # P36935 |
| RNAeasy Microisolation Kit | Qiagen | Cat # 73934 |
| Superscript IV Vilo | Thermofisher | Cat # 11756050 |
| RNAsin Plus | Promega | Cat # N2611 |
| Ribonucleoside vanadyl complex | Millipore Sigma | Cat # R3380 |
| RNase A | Sigma-Aldrich | Cat # R6148 |
| Neurotrace 640/660 | ThermoFisher | Cat # N21483 |
| ***Deposited data*** | | |
| Data supporting quantitative figure panels | Zenondo.org | https://zenodo.org/records/17113498 |
| ***Experimental models: Organisms/strains*** | | |
| *Khsrp^-/-^* mice | W.-J. Lin et al., 2011, Mol Cell Biol *31*, 3196-3207 | N/A |
| *Khsrp^fl/fl^* mice | S.L. Olguin et al., 2022, Commun Biol *5*, 672 | N/A |
| *Syn1-Cre* mice [B6.Cg-Tg(Syn1-cre)671Jxm/J] | The Jackson Laboratory | Strain #: 003966;  RRID: IMSR_JAX:003966 |
| C57Bl/J6 Mice | The Jackson Laboratory | Strain #: 000664  RRID:IMSR_JAX:000664 |
| Sprague Dawley Rats | Inotiv | Hsd:Sprague Dawley SD |
| **Oligonucleotides** | | |
| *Prenyl-Cdc42* 3’UTR nt 764-800 double stranded DNA oligonucleotide | Integrated DNA Technologies | NCBI Accession # XM_008764286.3 |
| *Prenyl-Cdc42* 3’UTR nt 764-838 double stranded DNA oligonucleotide | Integrated DNA Technologies | NCBI Accession # XM_008764286.3 |
| *Prenyl-Cdc42* 3’UTR nt 801-875 double stranded DNA oligonucleotide | Integrated DNA Technologies | NCBI Accession # XM_008764286.3 |
| *Prenyl-Cdc42* 3’UTR nt 839-913 double stranded DNA oligonucleotide | Integrated DNA Technologies | NCBI Accession # XM_008764286.3 |
| *RhoA* 5’UTR + *HinD3* restriction site – forward (corresponding to rat *RhoA*, GenBank Accession # XM_006243699). | Integrated DNA Technologies | 5’ CCCAAGCTTTGAGTATAAAATAGCAACTCGGTCTTTTATAG 3’ |
| *RhoA* 5’UTR + *BamH1* restriction site - reverse (corresponding to rat *RhoA*, GenBank Accession # XM_006243699). | Integrated DNA Technologies | 5’ CGGGATCCCACTTATGAAGGTGCTGAAGAAACTC 3’ |
| *RhoA* 3’UTR + *Not1* restriction site -forward (corresponding to rat *RhoA*, GenBank Accession # XM_006243699). | Integrated DNA Technologies | 5’ GGGGCGGCCGCAGCCTTGTGAC 3’ |
| *RhoA* 3’UTR + *Xho1* restriction site - reverse (corresponding to rat *RhoA*, GenBank Accession # XM_006243699). | Integrated DNA Technologies | 5’ GGGCTCGAGTTTAGAAAACTGCCT 3’ |
| *Prenyl-Cdc42* sense PCR primer | Integrated DNA Technologies | 5’ CGTTTGTGGGGATTTGCGTT 3’ |
| *Prenyl-Cdc42* antisense PCR primer | Integrated DNA Technologies | 5’ GACAGACGACCTGCACCTAC 3’ |
| *Prenyl-Cdc42* Taqman PCR probe | Integrated DNA Technologies | 5’ 56-FAM/  GCCCCCTTG/ZEN/CCCTTCCGGTA/3IABkFQ 3’ |
| *GFP* sense PCR primer | Integrated DNA Technologies | 5’ CTGCTGCCCGACAACCAC 3’ |
| GFP antisense PCR primer | Integrated DNA Technologies | 5’ TCACGAACTCCAGCAGGAC 3’ |
| *GFP* Taqman PCR probe | Integrated DNA Technologies | 5’ 56-FAM/ CCAGTCCGC/ZEN/CCTGAGCAAAGACC/3IABkFQ/ 3’ |
| *Gapdh* sense PCR primer | Integrated DNA Technologies | 5’ TGTGATGGGTGTGAACCACGAGAA 3’ |
| *Gapdh* antisense PCR primer | Integrated DNA Technologies | 5’ GAGCCCTTCCACAATGCCAAAGTT 3’ |
| Biotinylated *Prenyl-Cdc42* nt 764-838 RNA | Integrated DNA Technologies | NCBI Accession # XM_008764286.3 |
| Biotinylated *Prenyl-Cdc42* nt 801-875 RNA | Integrated DNA Technologies | NCBI Accession # XM_008764286.3 |
| Biotinylated *Prenyl-Cdc42* nt 764-800 RNA | Integrated DNA Technologies | NCBI Accession # XM_008764286.3 |
| Biotinylated Scramble RNA | Integrated DNA Technologies | N/A |
| Scramble siRNA (non-targeting) | Horizon Discovery Biosciences | Sense: 5'  CGUUAAUCGCGUAUAAUACGCGUA 3'  Antisense: 5'  UACGCGUAUUAUACGCGAUUAACG 3' |
| *Prenyl-Cdc42* siRNA | Horizon Discovery Biosciences | Sense: 5'  GCAAUGUUUAAAUCAAACUAAAGAU 3'  Antisense: 5'  AUCUUUAGUUUGAUUUAAACAUUGC 3' |
| *Khsrp* constitutive knockout mouse genotyping primer – forward (Khsrp forward P1) | Integrated DNA Technologies | 5’ TTCCGAAGCTCTGACTGGTC 3’ |
| *Khsrp* constitutive knockout mouse genotyping primer – reverse (Khsrp reverse P2) | Integrated DNA Technologies | 5’ CGGTGTTGTAGTCCGACATG 3’ |
| *Khsrp* constitutive knockout mouse genotyping primer – reverse (Khsrp reverse P2) | Integrated DNA Technologies | 5’ AAGGGTCCAGGGTTGAAAGG 3’ |
| *Khsrp^fl/fl^* mouse genotyping primer for inducible knockout – forward | Integrated DNA Technologies | 5’AGTGTTATGTGCTGGTGTGACCTGG3’ |
| *Khsrp^fl/fl^* mouse genotyping primer for inducible knockout – reverse | Integrated DNA Technologies | 5’GTGCTTACCCTTGACAGGGAGTGTC3’ |
| Syn1-Cre mouse genotyping primer – forward | Integrated DNA Technologies | 5’CTC AGC GCT GCC TCA GTC T3’  5’CAA ATG TTG CTT GTC TGG TG3’ |
| Syn1-Cre mouse genotyping primer – reverse | Integrated DNA Technologies | 5’GCA TCG ACC GGT AAT GCA3’  5’GTC AGT CGA GTG CAC AGT TT3’ |
| ***Recombinant DNA*** | | |
| GFP^MYR^3’prenyl-Cdc42^764-913^ plasmid | This paper | N/A |
| GFP^MYR^3’prenyl-Cdc42^914-2164^ plasmid | This paper | N/A |
| GFP^MYR^3’prenyl-Cdc42^764-800^ plasmid | This paper | N/A |
| GFP^MYR^3’prenyl-Cdc42^764-838^ plasmid | This paper | N/A |
| GFP^MYR^3’prenyl-Cdc42^801-875^ plasmid | This paper | N/A |
| GFP^MYR^3’prenyl-Cdc42^839-913^ plasmid | This paper | N/A |
| GFP^MYR^5’CamK2α/3’Actg plasmid | Willis et al., 2007, J Cell Biol *178*, 965-980 | N/A |
| GFP^MYR^5’/3’prenyl-Cdc42 plasmid | Lee et al., 2021, J Cell Sci *134*, jcs251967 | N/A |
| mCherry^MYR^5’/3’RhoA plasmid | This paper | N/A |
| ***Software and algorithms*** | | |
| Leica Application Suite X (LASX) software | Leica Microsystems | RRID:SCR_013673 |
| *NIH ImageJ* | https://imagej.nih.gov/ij/ | SCR_003070 |
| ImageJ Colocalization Plugin | https://imagej.nih.gov/ij/plugins/colocalization.html | N/A |
| GraphPad Prism 9 | https://www.graphpad.com/ | RRID: SCR_002798 |
| Clustal Omega | https://www.ebi.ac.uk/jdispatcher/msa/clustalo | RRID:SCR_001591 |
| JalView | https://www.jalview.org/ | RRID:SCR_006459 |
| ***RNA in situ hybridization probes*** | | |
| Q670 conjugated *prenyl-Cdc42* Stellaris Probe | BioSearch Technologies | Cat # SMF-1063-5 |
| Q570 conjugated *RhoA* Stellaris Probe | BioSearch Technologies | Cat # SMF-1063-5 |
| Q570 conjugated *GFP* Stellaris Probe | BioSearch Technologies | Cat # VSMF-1015-5 |
| Q670 conjugated Scramble Stellaris Probe | BioSearch Technologies | Cat # SMF-1065-5 |
| Q570 conjugated Scramble Stellaris Probe | BioSearch Technologies | Cat # SMF-1063-5 |
| Q670 conjugated *Dapb* Stellaris Probe | BioSearch Technologies | Cat # 802868854 |
