# Supplementary figures and images for: KHSRP-mediated decay of axonally localized prenyl-Cdc42 mRNA slows nerve regeneration
Source: PLoS Genet. 2025 Nov 7;21(11):e1011916. doi: 10.1371/journal.pgen.1011916 (PMC12614808; doi:10.1371/journal.pgen.1011916)

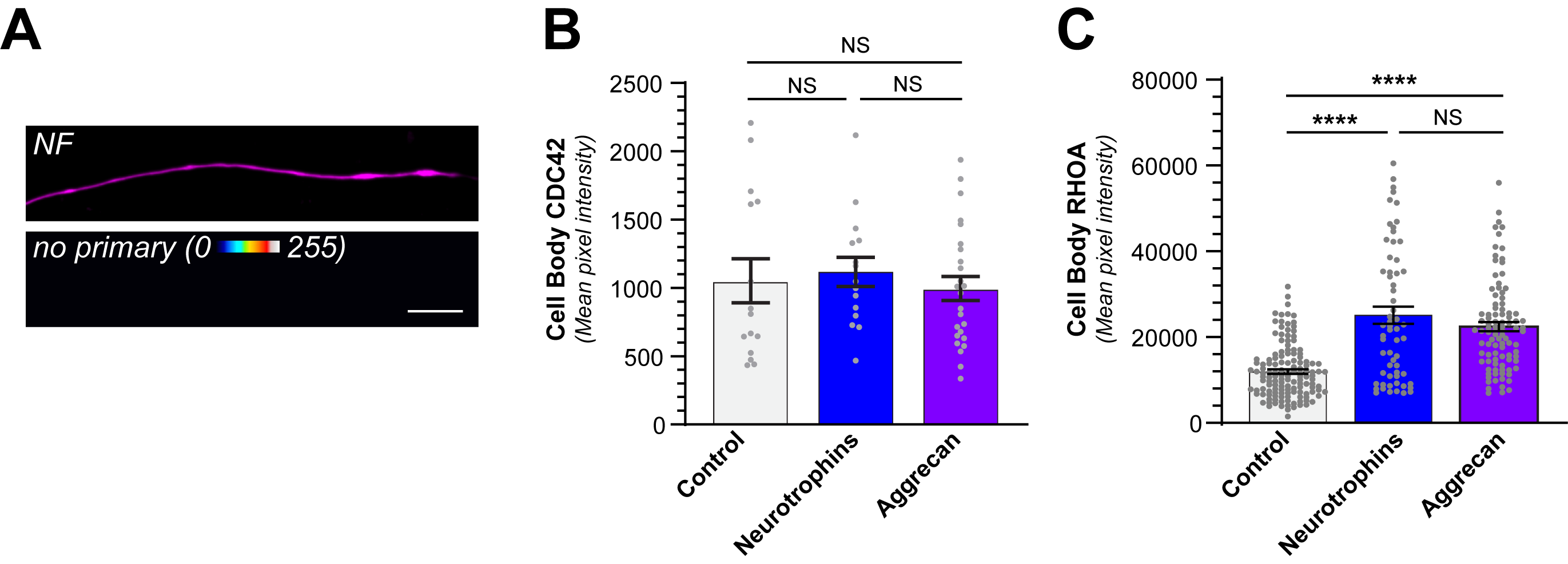

Supplement: S1 Fig — A) Representative IF images with no primary antibody as negative control for Fig 1D (see Fig 1E-F for quantifications). B) Quantitation of CDC42 signal intensities shown as mean pixel intensity above background ± SEM for cell bodies (N ≥ 15 neurons in three independent cultures; NS = not significant between indicated data pairs by ordinary one-way ANOVA with pair-wise comparison with Tukey post-hoc tests). C) Quantitation of RHOA signal intensities shown as mean pixel intensity above background ± SEM for cell bodies (N ≥ 15 neurons in three independent cultures; NS = not significant, **** P < 0.001 between indicated data pairs by Kruskal-Wallis ANOVA with pair-wise comparison with Dunn post-hoc tests). (TIF) [file pgen.1011916.s002.tif]

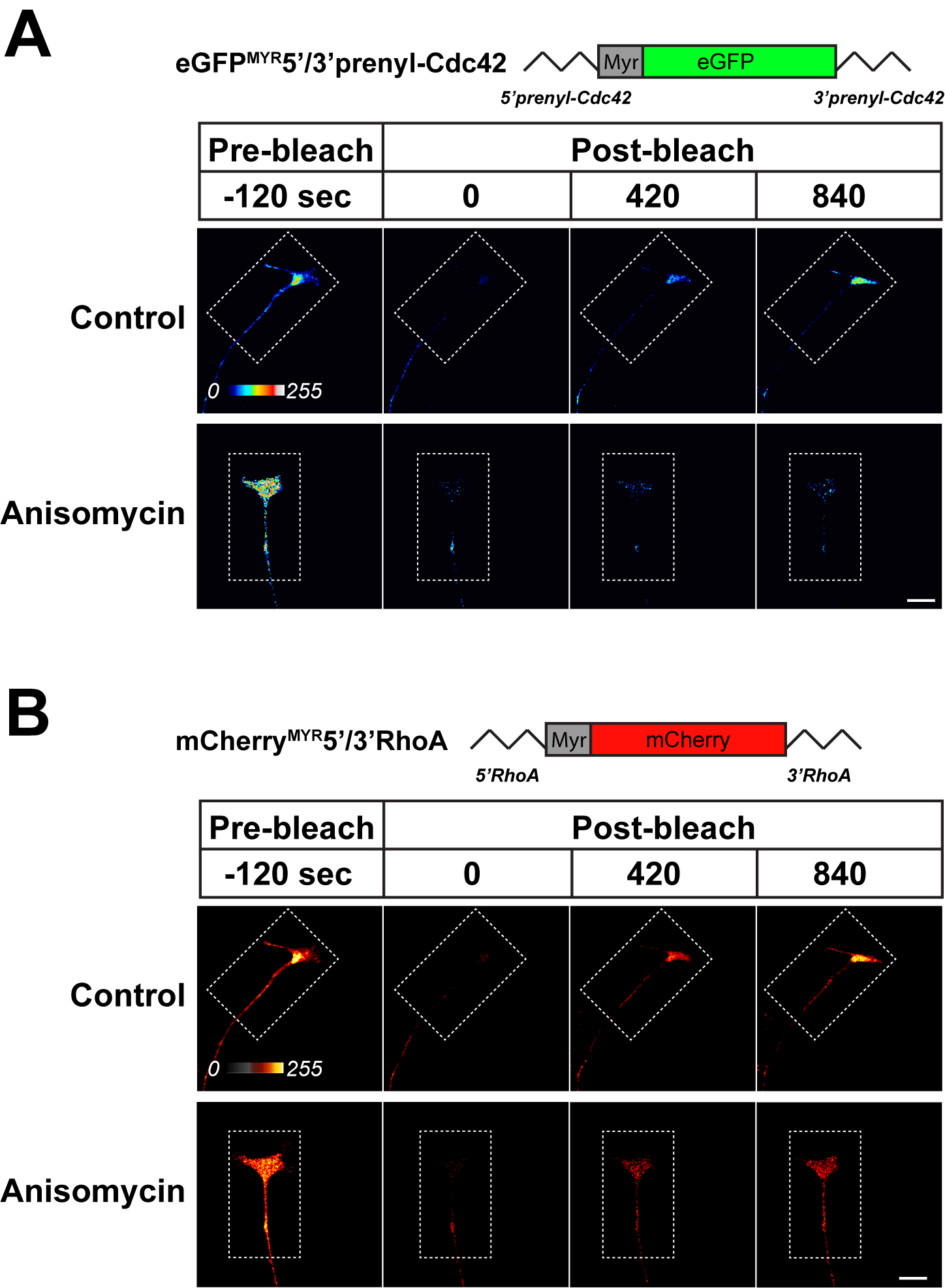

Supplement: S2 Fig — A-B) Representative FRAP image sequences for DRG neurons co-transfected with GFPMYR5’/3’prenyl-Cdc42 (A), and mCherryMYR5’/3’RhoA (B) at 72 h post-transfection are shown. Boxed regions represent the photobleached ROIs (see quantification in Fig 2B-C) [Scale bar = 20 µm]. (TIF) [file pgen.1011916.s003.tif]

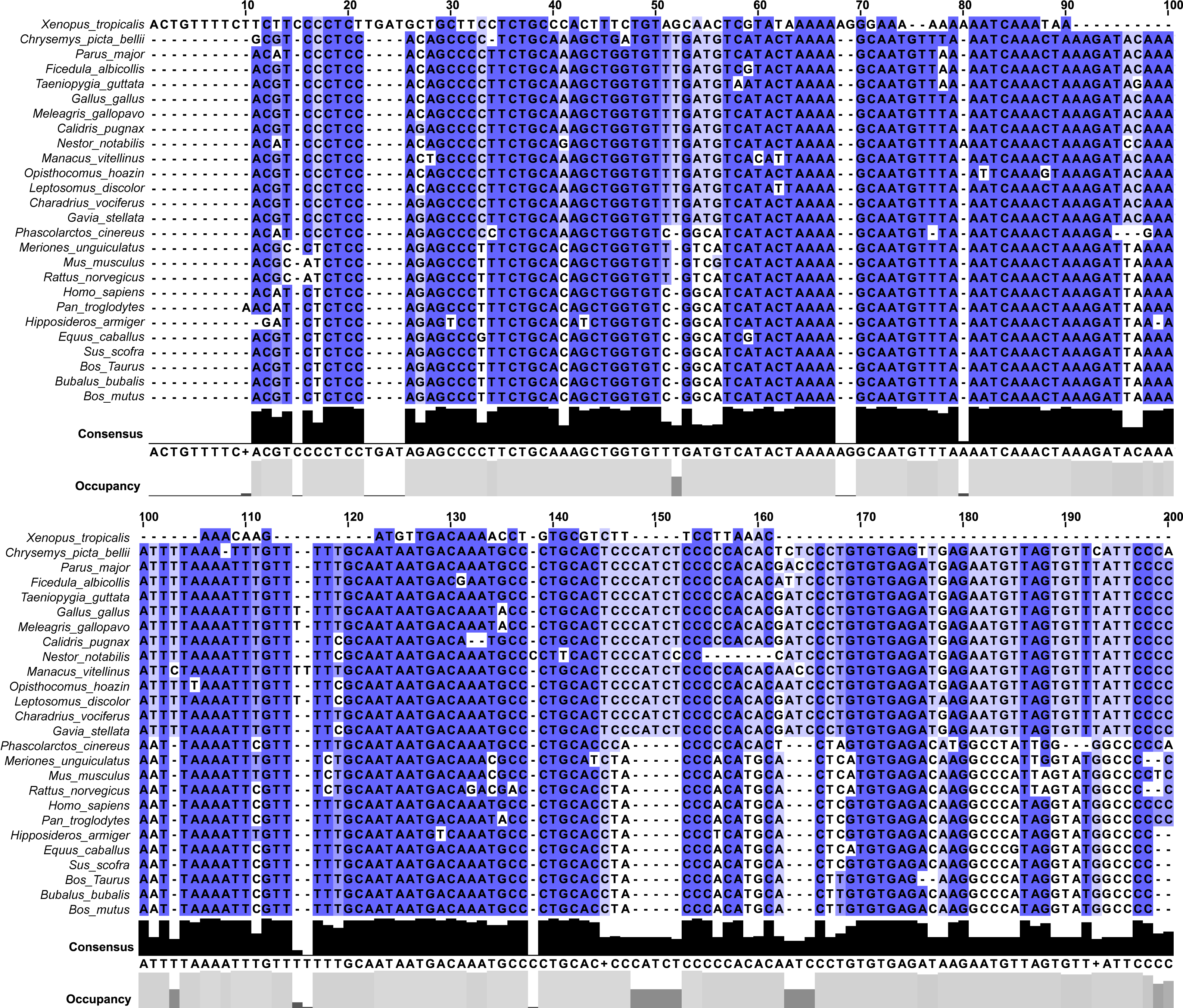

Supplement: S3 Fig — Clustal Omega multiple sequence alignment [55] for the 3’UTR of Prenyl-Cdc42 mRNAs are shown. Blue boxed regions show nucleotide conservation across orthologs. Nucleotide numbers labelled above start at the first nucleotide of the 3’UTR for Xenopus tropicalis. Beneath are graphical representations of consensus (% identity) and occupancy as well as a consensus aligned sequence. (TIF) [file pgen.1011916.s004.tif]

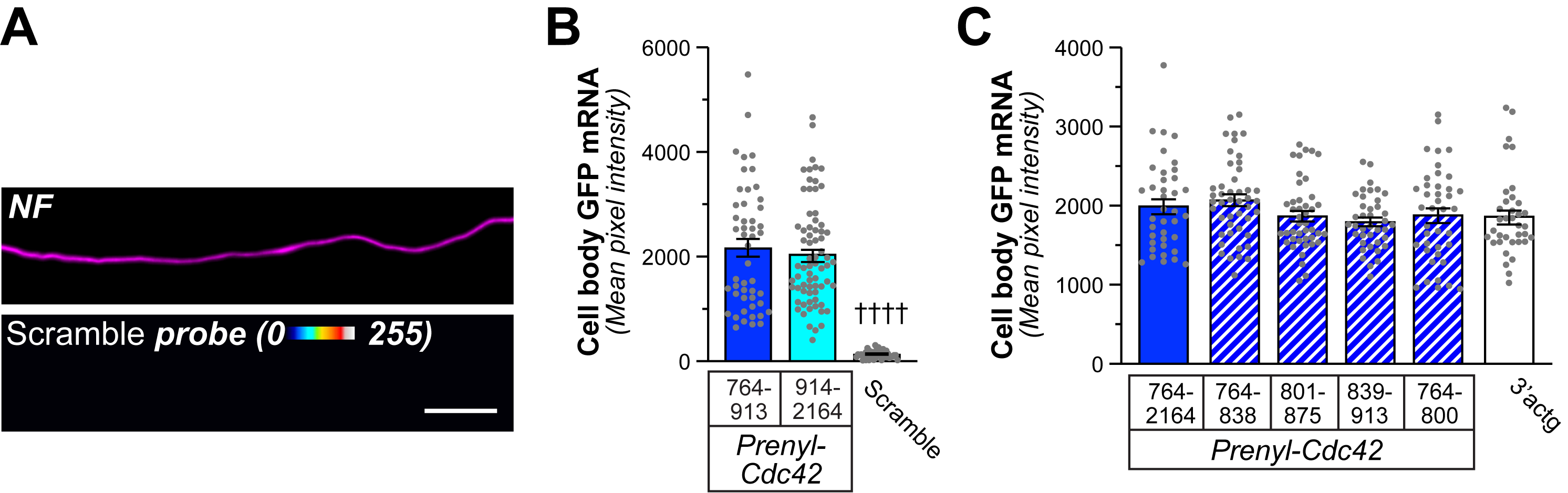

Supplement: S4 Fig — A) Representative smFISH images for scramble FISH probe as negative control exposure matched to those in Fig 3B & 3D (see Fig 3C & 3E for quantitative data) [Scale bar = 10 µm]. B-C) Quantitation of smFISH signal intensities shown as mean pixel intensity above background ± SEM for cell bodies corresponding to Figu 3D-E (N ≥ 40 neurons in three independent cultures; not significant between any data pairs by one-way ANOVA, pair-wise comparison with Tukey post-hoc tests). (TIF) [file pgen.1011916.s005.tif]

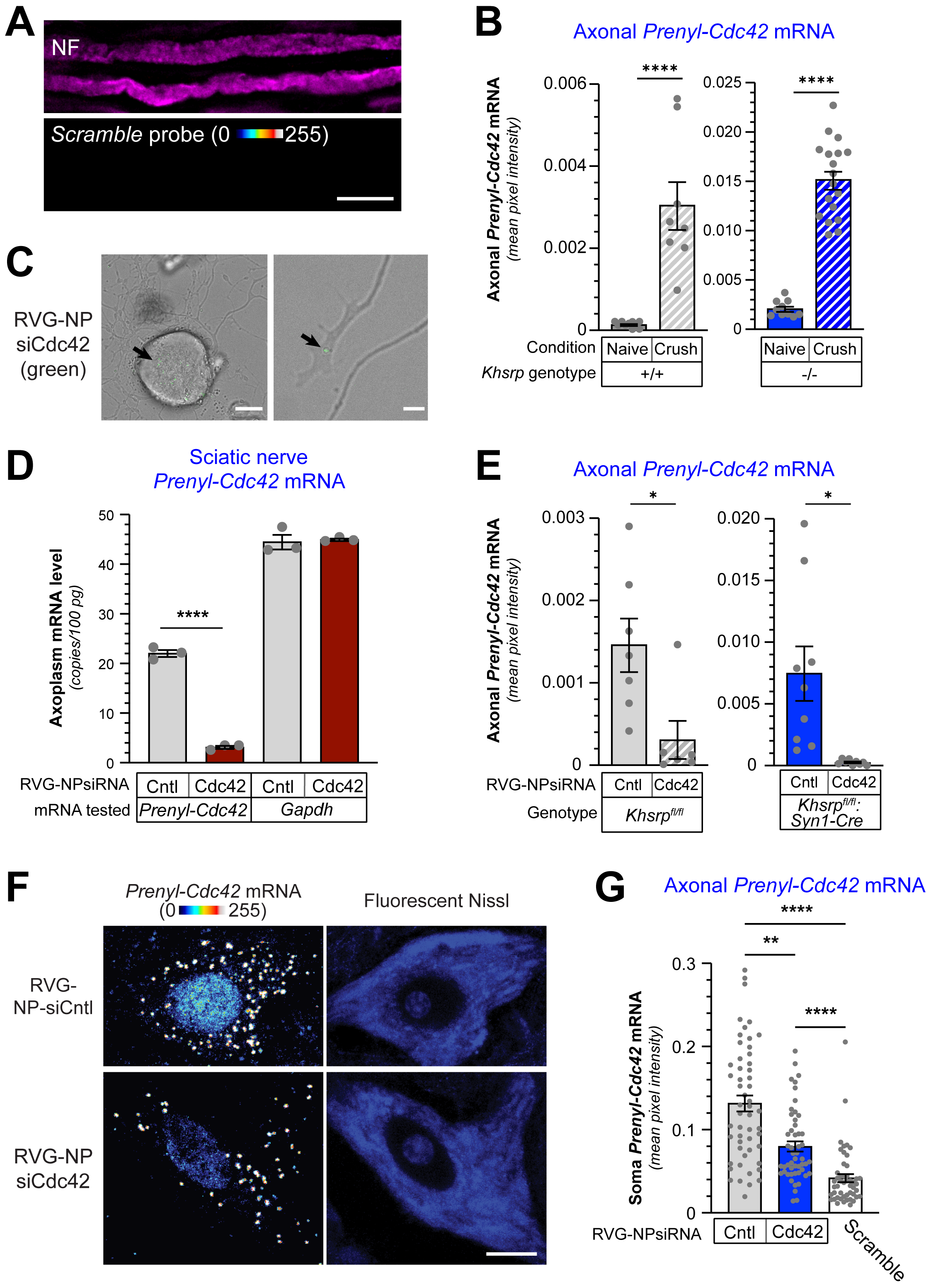

Supplement: S5 Fig — A) Representative smFISH images for 7 day post-crush injured sciatic nerve showing signals for scramble probe exposure matched to Fig 5A (see Fig 5B for quantitation) [Scale bar = 5 µm]. B) Quantitation of smFISH signals for RNA probe signals for individual Khsrp genotypes from Fig 5B as mean ± SEM (N = 3 biological replicates; **** P < 0.001 by Welch’s t-test for individual comparisons). C) Representative transmitted light image merged with signals for Alexa488-labeled RGV-NP-siCdc42 (Green) treated wild type mouse DRG cultures. Both the soma (left) and distal axon with growth cone (right) show apparent intracellular DiD signals (arrows) [Scale bar right panel = 25 µm, left panel = 10 µm]. D) Quantification of axoplasm Prenyl-Cdc42 and Gapdh mRNA levels from wild type mice injected with RVG-NP-shCntl vs -siCdc42 (N = 3 animals per condition; **** P < 0.001 by Welch’s t-test for individual comparisons). E) Quantification of smFISH for axonal Prenyl-Cdc42 mRNA levels from Fig 5B separated as Khsrpfl/fl and Khsrpfl/fl:Syn1-Cre mice treated with RVG-NP-siCntl vs. -siCdc42 as mean ± SEM (N = 3–5 animals per condition; * P < 0.01 by Welch’s t-test for individual comparisons). F-G) Representative exposure matched smFISH images for Prenyl-Cdc42 mRNA + Nissl substance (F) and motor neuron smFISH signal quantitation (G) for Khsrpfl/fl:Syn1-Cre mice that had received RVG-NP-siCntl vs. -siCdc42 nerve injections 5 days prior to euthanasia (N ≥ 40 neurons, N = 3 animals; ** P < 0.01 and **** P < 0.0001 between indicated data pairs by Kruskal-Wallis ANOVA with pair-wise comparison with Dunn post-hoc tests) [Scale bar = 10 µm]. (TIF) [file pgen.1011916.s006.tif]

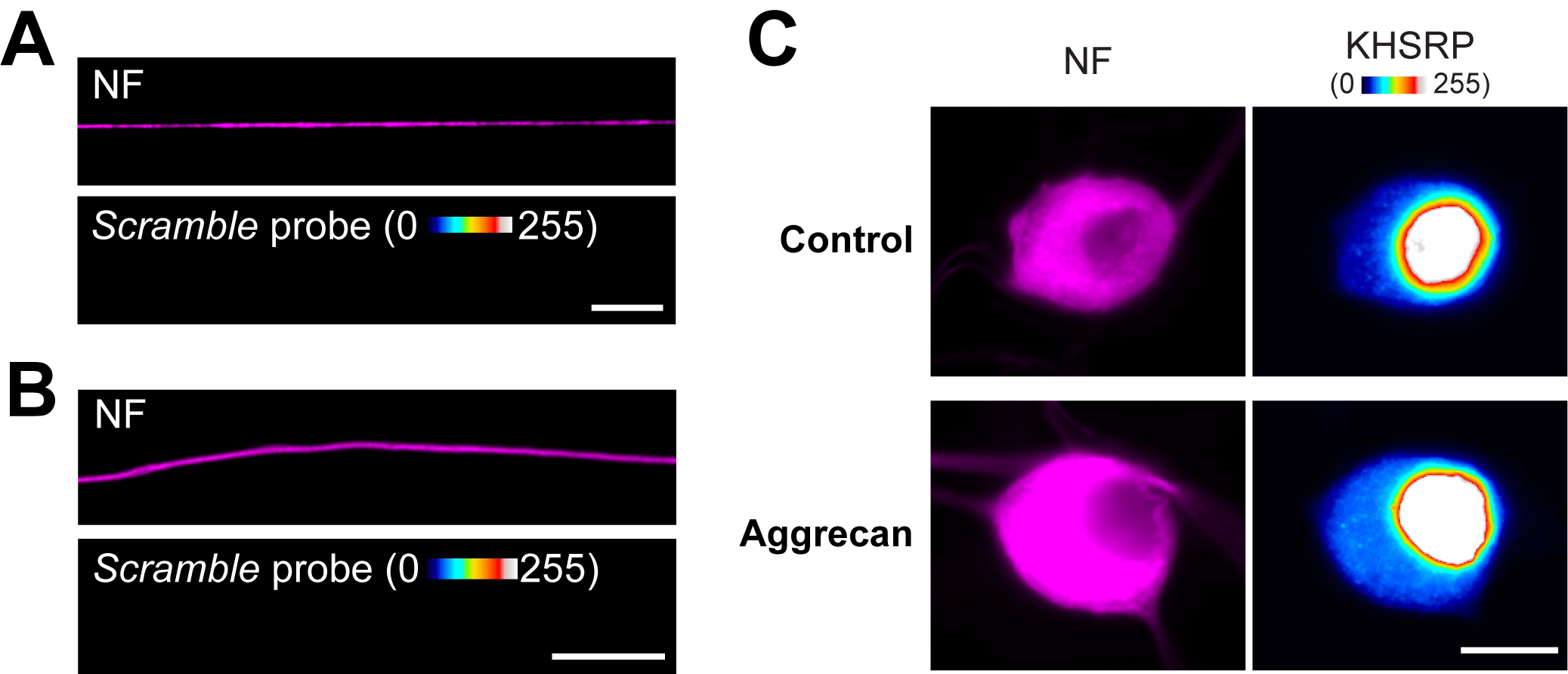

Supplement: S6 Fig — A) Representative smFISH images using scramble probe as negative control in adult mouse Khsrp-/- DRG neuron cultures exposure matched to Fig 6A (see Fig 6B for quantitation) [Scale bar = 10 µm] B) Representative smFISH images using scramble probe as negative control in adult mouse DRG neuron cultures exposure matched to Fig 6C (see Fig 6D for quantitation) [Scale bar = 10 µm]. C) Representative exposure matched IF images for KHSRP protein in cell bodies of cultured mouse DRG neurons exposed to aggrecan as in Fig 6E (see Fig 6F for quantitation) [Scale bar = 25 µm]. (TIF) [file pgen.1011916.s007.tif]
